# Where Is My Mind…? The Link between Mind Wandering and Prospective Memory

**DOI:** 10.3390/brainsci12091139

**Published:** 2022-08-26

**Authors:** Jean-Charles Girardeau, Marco Sperduti, Philippe Blondé, Pascale Piolino

**Affiliations:** 1 Laboratoire Mémoire, Cerveau et Cognition (LMC2 UR7536), Institut de Psychologie, Université Paris Cité, 92100 Boulogne-Billancourt, France; 2 Laboratoire de Psychologie Sociale et Cognitive (LAPSCO), Université Clermont Auvergne, 63000 Clermont-Ferrand, France

**Keywords:** prospective memory, mind wandering, experience sampling

## Abstract

Mind wandering (MW) is a common feature of the human experience occurring when our attention shifts from the task at hand to inner thoughts. MW seems to be often future-oriented and could be used to help people to carry out their planned actions (Prospective Memory PM). Here, we tested the link between MW and the ability to perform PM intentions. We assessed MW and PM over 15 days using experience-sampling probes via mobile phone (226 participants) associated with a naturalistic PM task. We confirmed that MW occupies a significant proportion of our mental activity (40%). This time seems to be mainly used to project ourselves into the future (64%), whether to anticipate and imagine the long term (20%) or to plan daily obligations (44%). Intriguingly, only past-oriented thoughts (9%) predict the PM performance. We discuss the possible functional role played by MW in maintaining intentions in mind.

## 1. Introduction

While reading the present paper, you may experience a situation in which your eyes pass over the lines of a paragraph, but your mind is focused elsewhere. Hence, although you are physically reading, you are not paying attention. This phenomenon, known as mind wandering (MW; [1,2], is a fairly common experience occupying between 30% and 50% of our waking life [3,4,5]. The core feature of MW is an attentional disengagement from processing the environment to focus on internal thoughts. Thus, when MW, external stimuli fade from conscious perception in favor of internal ones, a phenomenon called “perceptual decoupling” [6]. Although this anecdotal description of MW is easily relatable, its operationalization and theoretical definition have been heavily debated [7,8,9]. Some of the main characteristics used to characterize MW are task-unrelated thoughts [2,10], stimulus-independent thoughts [11,12], or a dynamic and unconstrained flow of thoughts [13]. Furthermore, it has been suggested to distinguish two forms of MW: spontaneous and voluntary [14,15]. The former corresponds to an involuntary switch from a task-related focus to a task-unrelated one, while the latter represents a conscious choice to stop paying attention to the task at hand. This distinction highlights two different causes for MW: spontaneous MW seems to be the consequence of executive control failure [16] and is associated with high distractibility [17,18], while voluntary MW seems more tied to a diminished motivation [19,20].

Whichever definition is used, the core of MW is an attentional disengagement from the environment, which is not a phenomenon without consequences. Several reviews underlie how MW can impact the functioning of various cognitive processes [21,22,23,24]. The overall consensus is that it has a broadly negative impact, hindering focused attention [25,26], working memory capacity [27,28] and episodic memory encoding [29] to name a few. However, it would be curious if such a prevalent phenomenon served no purpose. Some experimental results suggest that MW can also lead to positive outcomes [21]. One of the more intriguing is that particular instances of MW could help improve memory encoding [30,31,32]. Notably, stimulus-dependent thoughts (i.e., thoughts associated with an environmental cue but not related to task resolution) can facilitate encoding by allowing for extra elaboration of the stimulus [31,32]. In addition to memory encoding, it has also been suggested that MW may also enhance memory consolidation. Indeed, recalling a memory has been shown to result in a re-encoding of the event, promoting its long-term consolidation [33,34]. Therefore, MW could be instrumental in the retention and distinctiveness of long-term memories by regularly retrieving these memories and re-encoding them [35]. Moreover, one likely adaptive function of MW could be to disengage cognition from the here and now to envision future goals. Several studies have reported that MW is mostly future-oriented and directed toward completing future self-relevant goals [12,36,37]. Accordingly, it has been suggested that MW is characterized by an autobiographical bias [36] and could be triggered by the current concerns of an individual [38]. More precisely, future-oriented MW would serve more the successful completion of particularly concrete goals (i.e., planned intentions), rather than less concrete episodic future thinking [39]. Surprisingly, very few studies have directly tested the link between the propensity to MW and prospective memory (PM).

PM consists of remembering to carry out an action that was previously planned, at some point in the future [40,41,42]. From planning to execution of an intention, the whole PM process is underpinned by various cognitive processes and requires attentional and executive resources [43,44,45,46,47]. The triggering of PM intention recovery, involving either automatic or controlled processes or both [42,48], could be triggered by external cues (*event-based*) or can be auto-initiated (*time-based*) when the action must be performed after a specific time, without external help. Unlike retrospective memory, on which PM relies in part, PM does not simply store memories or knowledge *per se*, but the intention to perform an action at the appropriate time or context. This means that the PM encoding process is twofold, including the formation of an action (e.g., I have to buy bread) and the moment at which it must be executed (e.g., when coming home to work). In between, the intention is set in the background for a more or less long period. During this delay, ongoing activities may capture attention and interfere with the execution of the PM intentions, both tasks relying on the same processes [49]. So, opportunities to complete intended tasks often require multitasking [50,51]. Yet, how people manage the competition between PM and ongoing-task demands could partly be under metacognitive control [52].

As searching the PM target may rely on primarily controlled or automatic processing, it is important that one deploys their attentional resources strategically. Metacognition would explain the observed variation in PM costs by assuming that the awareness of PM-demand controls to what extent people rely on strategic versus spontaneous PM retrieval. The metacognitive top-down control process of PM could be based both on metacognitive expectations about the PM task and good metacognitive awareness of contextual PM demands [53]. Thus, participants having good metacognitive awareness of the situations would better coordinate PM monitoring [48,54]. This is precisely what Seli et al. [55] recently reported through the observation of a positive link between the rate of self-caught MW and the monitoring of PM time-based goal, highlighting a plausible common monitoring system for the content of consciousness in general and PM intentions. In the same vein, Girardeau et al. [56] compared the effect of mindfulness and an MW induction, two supposedly opposed cognitive states, on PM. Intriguingly, they did not report any difference between the two conditions on PM performance, even if the two inductions subjectively had a differential effect on the participants’ cognitive state. It must be noted that the MW induction required participants to mind wander voluntarily. Intentional episodes of MW have been strongly associated with planning and the awareness of the initiation of the episode of MW, resulting in a meta-cognitive awareness of its occurrence [15]. Given the link between meta-awareness and PM, one likely explanation is that MW could have had a similar beneficial effect on PM performance via a transient increase in PM monitoring. All these data seem to fit well with the theoretical account [9,55,57] stating that meta-awareness includes a monitoring system intermittently “checking” consciousness, thereby allowing people to consider the relation of the current contents of consciousness and goals. This description fits particularly well with PM tasks.

So far, MW has been mainly associated with attentional and memory failures. A general finding now emphasizes the existence of a “prospective bias” in MW as off-task thoughts are more frequently categorized as being about the future. If disengaging from a primary task can help one to accomplish a desired future goal, then these attentional shifts could, in fact, be constructive. MW episodes would thus be the given opportunity for a latent PM goal, according to attentional demand, to become active in the mind and to be accomplished. Furthermore, the possible common monitoring system that would monitor consciousness content and both PM goal and ongoing task progress [9], would thus maintain a balance between the content of consciousness and goals [57]. This ability to monitor would highly depend on individual differences in metacognitive mind-wandering abilities and individual differences in the ability to maintain continuous goal monitoring [55]. Lastly, people make implicit metacognitive judgments about how difficult the target condition will be to detect and alter their attentional allocation accordingly [58]. For this reason, it would be relevant to study if the MW dimensions, content or source, influence this metacognitive perception, and in turn, PM performance.

Even if this has been widely theorized that letting your mind wander could offer advantages for retrieval of PM intentions, to our knowledge, except for Girardeau et al. [56] who used MW as a control condition in a study on mindfulness, no study has investigated the direct link between the nature of MW and PM in a single study. Therefore, the objective of this study, using combining experience-sampling via mobile, was to investigate the link between the content and the type of MW and the ability to perform PM intentions. More precisely, we wanted to study the link between what individuals do, what they think about while doing it, and their ability to respond to a naturalistic PM task. We will then observe whether the tendency towards voluntary or spontaneous MW, the temporal orientation of MW (past/future), and the interaction between these features predict the execution of prospective intentions. Based on respective research on MW and PM [4,55,59,60], we argue that individuals not only spend more time thinking about the future rather than the past, and this deliberately, but that these future thoughts are directly related to the planning and execution of PM intentions. Additionally, engaging in a deliberate form of prospective-oriented MW will lead to better PM performance compared to engaging in a spontaneous prospective-oriented one.

To answer this question, the use of experience-sampling, via smartphone technology, offers a double advantage. First, it allows including a large number of participants and the assessment of MW under ecological conditions [4]. Secondly, it is particularly useful for investigating spontaneous PM-related thoughts over long retention intervals outside the laboratory, more specifically for addressing the question of whether prospective bias in MW predicts concurrent completion of a PM task. We restricted our investigation to time-based PM for methodological and theoretical reasons. Firstly, event-based tasks are harder to operationalize and control in an ecological setting. Secondly, the theoretical link between MW and time-based PM seems best supported by the previously mentioned study [55]. Naturalistic methodologies to study MW have long combined experience-sampling and thought probes methods [5,12,61,62]. In contrast, the amount of research using naturalistic PM assessments is relatively modest. Nevertheless, PM research seems to be moving towards naturalistic PM tasks [59,63,64,65,66], certainly because of the diffusion of mobile devices, in particular smartphones. These PM experience-sampling studies are inspired by those conducted to study the MW. The novelty of our methodology was to combine the long-lasting tradition of MW experience-sampling methods and the renewal of PM naturalistic tasks.

## 2. Materials and Methods

### 2.1. Procedure

#### 2.1.1. Participants

Three hundred and forty-two participants took part in this study. The exclusion criteria concerned all persons under 18 years of age and over 65 years of age, non-French speaking and with a neurological or psychiatric history. We had to deal with an important loss of subjects due to a variety of reasons such as withdrawal, incomplete identification of subjects or technical bugs (11 participants excluded). In addition to this experimental mortality, we excluded all participants who completed less than half of the thought probes (85 participants excluded). Lastly, PM is a cognitive function whose performance declines with aging. Typically, the development of PM abilities follows an inverted U-shaped curve that results in an increase in performance until age 20, followed by a gradual decrease throughout adulthood, especially after age 65 [67]. So, we exclude participants over 65 years old in order to avoid any experimental bias related to age. After data cleaning, this study concerned 226 participants (mean age 37.85 ± 12.88; 180 female, 45 male, 1 other).

All participants were recruited by advertisement on various social networks such as LinkedIn, Facebook, Instagram and Risc (Relay of information on the sciences of cognition). Recruitment was performed via a web link provided in the study announcement. The webpage behind the link contained all the information concerning the nature and objectives of the study, its conduct and its potential risks. It also included the declaration of consent and a shared liability clause. Lastly, before the end of the subscription, participants were submitted to a brief anamnesis to collect socio-demographic information (age, gender, education, meditation expertise).

This project was approved by the Research Ethics Committee of Université Paris Cité. The different tools used in this study met the requirements of the General Data Protection Regulation (GDPR).

#### 2.1.2. Materials

All surveys were created and administered using LimeSurvey, a free and open-source online survey application. Text message signals were sent to participants’ phones (each containing a link to a survey) using Sendinblue, a French Digital/Marketing Automation platform.

### 2.2. Overall Conduct

**Figure 1:**
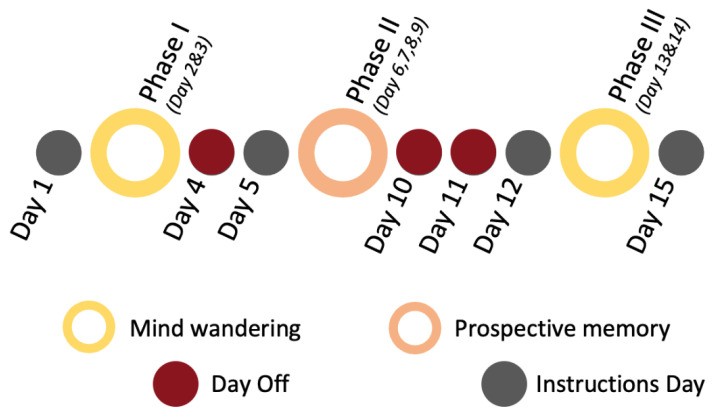
Overall conduct of the study process.

The study took place over 15 days and was divided into three parts (Figure 1). To start (Day 1), participants received the first text message, containing a link to the thought probing phase instructions and the MPMI-s. During the 1st and 3rd parts (Days 2 to 3, and 13 to 14), devoted to mind wandering assessment (Phase I—*at the beginning* and Phase III—*at the end of the study*), participants received a series of text messages inviting them to join an online questionnaire, containing 7 multiple answer questions. Just before Phase II begins (Day 5), participants received a single text message with a link to the prospective memory phase procedure instructions (Phase II). In particular, the procedure contained the link that they used to report the execution of their PM task. During Phase II (Days 6, 7, 8, 9), participants had to perform the same PM task every day over 4 days (Phase II—*Prospective Memory Phase*). Before starting Phase III (Day 12), a single text message containing a link to a reminder of the thought probes phase procedure instructions (identical to Phase I) was sanded to the participants to inform them that it will begin the day following receipt of this message. A final text message was sanded to the participants at the end of the experiment (Day 15), containing a link to a final survey, where they were suggested to complete two last questionnaires about mind wandering dispositional (MWQ) and anxiety/depression (PHQ4), and provided with additional information on the objectives of the study. Some days are missing in this procedure (4, 10, 11), this is mainly due to the prohibition of cold calling on Sundays. In consequence, once registered, participants never started the first part of the study until the following Thursday, so that no phase was interrupted by a day off.

### 2.3. Experience Sampling

#### 2.3.1. Mind Wandering Assessment

During Phases I and III, corresponding to the thought probes phases (Mind wandering), six text messages were sent each day, bringing the total number of possible surveys to 24 per participant (Table 1). Text messages occurred pseudo-randomly between 8 a.m. and 10 p.m., with the constraint that two probes could not be delivered with a delay shorter than 1 h. We intentionally kept the number of surveys low enough to not be considered a burden, and more importantly, so participants would not be thinking about the study all the time. A minimum of 12 completed surveys was required to be included in the subsequent analysis. Participants were encouraged to respond immediately upon seeing the SMS; however, should they find themselves in the middle of an important or delicate activity, such as driving a car, for example, they will be asked not to take the survey. By using these procedures, we sought to minimize the number of incomplete surveys, without requiring participants to rely too much on their long-term memory. For each survey, questions remained in the same order (Table 2).

Though we were primarily interested in the frequency of thoughts related to PM, special care was taken not to bias participants, and each of the seven questions was given equal consideration, emphasis, and explanation.

Each participant received a total of 24 thought probes (4 × 6). For each probe, the participant had the choice between Focus (on his ongoing task) or Mind wandering (declined in detail, i.e.*, episodic future thinking (EFT), Planning, episodic past thinking (EPT), Imagination, Nothing*). For each participant, we calculated an overall mind wandering frequency by dividing the number of responses off-task (MW) by the total number of probes answered by the participant (Focus and MW). We then applied the same calculation to compute the frequency of each content of mind wandering: *episodic future thinking (EFT), Planning, episodic past thinking (EPT), Imagination, Nothing*. In the same way, we computed the frequency of each type of mind wandering: *spontaneous, external, voluntary*.

#### 2.3.2. Prospective Memory Assessment

During the 4 days of Phase II, corresponding to the prospective memory assessment, participants were invited to perform the same action every day (click on a link at 3:30 p.m.). The aim here is to measure the time-based capacities. We explicitly asked them to refrain from using external memory aids (for example, a phone notification) and instead try to implement the intention using only their “natural memory”. At the target time, participants had to go to an online survey, thus signaling the execution of this action, and had to answer 5 questions examining the context of recalling the intention (Table 3).

We considered as correct responses those given within a time window of 1 h (30 min before and 30 min after) around the target time. We then divided each of these scores by 4 (the total PM actions) to obtain a correct response, an incorrect response and an omission ratio.

#### 2.3.3. Questionnaires

We assessed our participants’ prospective memory abilities and strategy use with the short version of the Metacognitive Prospective Memory Inventory (MPMI-s—[68]). It consists of three 5-point Likert response scales ranging from 1 (Rarely) to 5 (Often), with eight items each and evaluating individuals’ perceptions of their own prospective memory abilities (e.g., “If I’ve borrowed something from someone for a while, I remember to give it back to that person the next time we see each other”), their frequency of use of internal prospective memory strategies to help themselves remembering (e.g., “In my mind I make a list of things that I still have to complete”), and external prospective memory strategies to better remember their intentions (e.g., “I write shopping lists”). In each scale, scored on 8 points, higher scores indicate better prospective memory abilities or more frequent strategy use.

We also used the Mind Wandering Questionnaire (MWQ; [28]), a self-report 5-item questionnaire that evaluates our participants’ natural tendency to experience episodes of mind wandering. It is composed of 5-point Likert response scales ranging from 1 (Almost never) to 5 (Almost always). The higher the score, the greater the propensity to wander.

Finally, we included the “Patient Health Questionnaire-4” (PHQ-4; [69,70]), a 4-item inventory useful for detecting cues of both depression and anxiety. It is composed of 4-points Likert response scales ranging from 1 (Never) to 4 (Almost every day). The higher the score, the greater the depressive and anxious traits. Its purpose is to allow for a very brief evaluation of depression and anxiety disorders. This questionnaire was included in order to nuance our results, participants presenting high depressive and anxious traits could present a different pattern of mind wandering [71,72,73,74] than healthy subjects.

### 2.4. Data Analysis

All statistical analyses were conducted with JASP (0.11.1) to test our different hypotheses. We conducted a repeated measure ANOVA on the ratio of MW with two factors: the content of MW (EFT, Planning, EPT and Imagination) and the type of MW (Spontaneous, External, Voluntary). The post-hoc analyses were corrected using the Bonferroni correction. Two multiple linear regressions were performed to predict the PM score with rates of the different components of mind wandering (content and source) and different independent measures collected with the questionnaires (prospective metamemory, mind wandering trait). We also included some covariates that may influence predictions such as Age and Mood (Depression and Anxiety). We reported the effect size for ANOVAs with η^2^_p_ (partial eta squared). A standard statistical significance level of 0.5 was used.

## 3. Results

### 3.1. Consistency of Mind Wandering’s Measure

The reliability of our experience-sampling method of mind wandering is illustrated by the observation of a positive relationship between the MW trait, measured by the MWQ, and the MW frequency, measured by the thought probes, r = 0.232, CI [0.076; 0.377], *p* = 0.004 (see detailed statistics in Supplementary Material S1).

**Figure 2:**
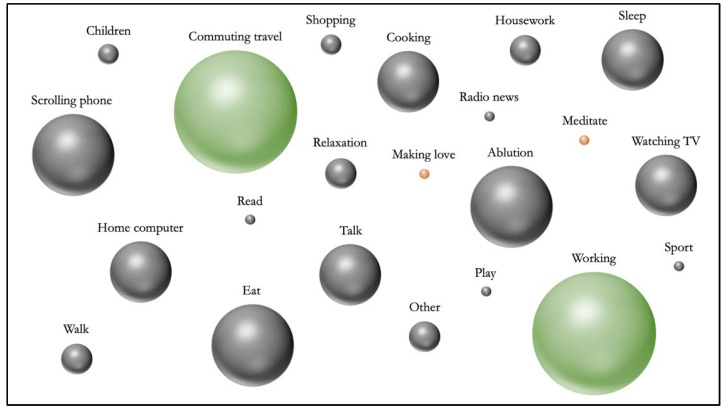
Frequency of different activity during MW episodes.

Calculation of the number of mind-wandering episodes in relation to the number of surveys answered (frequency calculation) showed that mind wandering occurred in 39.6% ± 0.168 of thought probes answered (4465). Mind wandering seems to take place more while people are working (15.81%). Conversely, the activity during which people experience fewer episodes of mind wandering is making love (0.11%) (see Figure 2 and Table 4).

**Figure 3:**
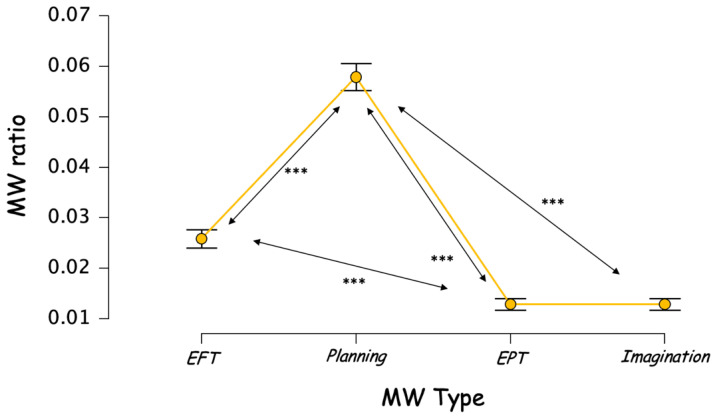
Frequency of different MW contents, calculated on all thought probes. *** *p* < 0.001.

We observed a significant main effect of the content, F(3,675) = 189.661, *p* < 0.001, ƞ_p_^2^ = 0.457 (Figure 3). A post-hoc analysis, with Bonferroni correction, revealed that the content of the mind wandering episodes was significatively more planning-oriented (17.4%, marginal mean = 0.058, 95% CI [0.053; 0.063]) than EFT-oriented (7.8%, marginal mean = 0.026, 95% CI [0.023; 0.029]), EPT-oriented (3.9%, marginal mean = 0.013, 95% CI [0.011; 0.015]), and Imagination-oriented (4.9%, 0.013, 95% CI [0.011; 0.015]). EFT-oriented thoughts were more frequent than EPT-oriented (mean_difference_ = 0.013, 95% CI [0.007; 0.019]), or Imagination-oriented (mean_difference_ = 0.013, 95% CI [0.007; 0.019]). The latter two being not significantly different (mean _difference_ = 3.979^−16^, 95% CI [−0.006; 0.006]).

**Figure 4:**
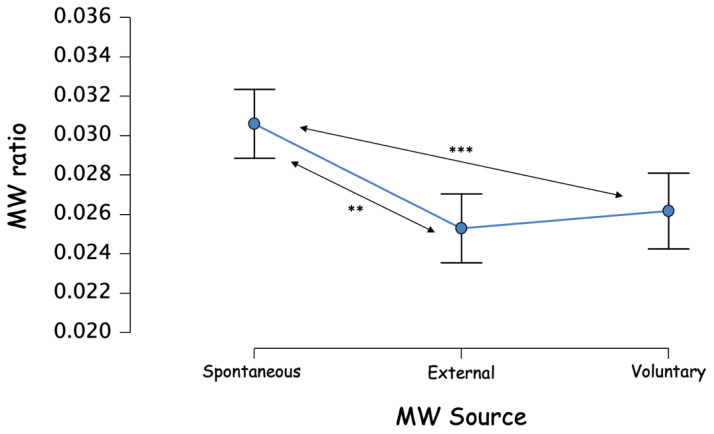
MW source frequency, calculated on all thought probes. ** *p* < 0.01, *** *p* < 0.001.

There was also a significant main effect of the MW source, F(2,450) = 6.520, *p* = 0.002, ƞ_p_^2^ = 0.028 (Figure 4). A post hoc analysis, with Bonferroni correction, showed that the source of the mind wandering episodes was more Spontaneous (0.031, 95% CI [0.027; 0.034]) than External (0.025, 95% CI [0.022; 0.029]) and Voluntary (0.026, 95% CI [0.023; 0.030]). The latter two being not significantly different (mean_difference_ = −8.823, 95% CI [−0.007; 0.005]).

**Figure 5:**
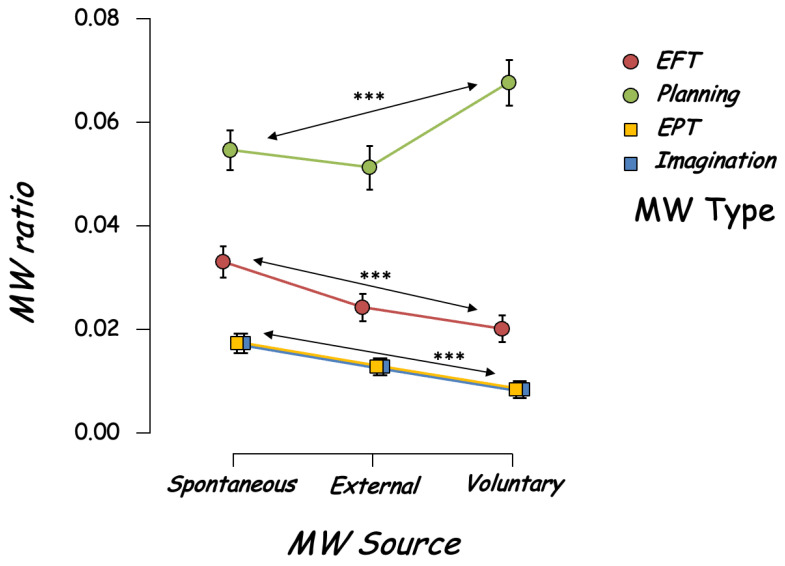
Interaction between MW contents and source frequency. *** *p* < 0.001.

Lastly, we reported a significant interaction between the MW content and the MW source, F(6,1344) = 2.386, *p* < 0.027, ƞ_p_^2^ = 0.011 (Figure 5). Post hoc analyses showed that the observed interaction is due to the fact that for all MW sources, except for planning-oriented thoughts, MW was more Spontaneous than External and Voluntary (see detailed statistics in Supplementary Material S2). On the contrary, planning-oriented thoughts were more voluntary (0.068, 95% CI [0.059; 0.076]) than spontaneous (0.055, 95% CI [0.047; 0.063]) and external (0.051, 95% CI [0.043; 0.060]).

### 3.2. Consistency of Prospective Memory’s Measure

We conducted a backward hierarchical regression on the PM correct ratio of prospective memory responses to verify the reliability of our prospective memory task. We entered as predictors the three components of Prospective metamemory: Aptitude, Internal strategy and External strategy. As age, depression and anxiety may impact PM performance, we included these factors as covariates in the regression. We only observed a positive relation between Aptitude and the PM correct ratio. The Aptitude component significantly predicted better PM performance, t(136) = 2.618, *p* = 0.010, 95% CI [0.002; 0.015], β = 0.220. The global model was significant, F(3,136) = 6.852, *p* = 0.010. R^2^ = 0.048.

### 3.3. Predictors of PM Correct Ratio

We conducted another backward hierarchical regression on the PM correct ratio of prospective memory responses with the following predictors: MWQ, Global MW frequency, ratio of MW of each Type (EFT, Planning, EPT, Imagination) and Source (Spontaneous, External, Voluntary). Meta Aptitude, Age, Depression and Anxiety were used as covariates in the regression.

The Meta Aptitude still predicted the PM correct ratio t(136) = 2.099, *p* = 0.038, 95% CI [5.295^−4^; 0.018], β = 0.172. We also reported that the “trait” of mind wandering, measured by the MWQ questionnaire, negatively and significatively predicted PM correct ratio, t(136) = −2.123, *p* = 0.036, 95% CI [−0.027; −9.402^−4^], β = −0.174. Moreover, episodes of past-oriented mind wandering significatively and positively predicted PM correct ratio, t(136) = 2.280, *p* = 0.024, 95% CI [0.065; 0.919], β = 0.186,. No other MW content predicted the PM correct ratio. We reported that the global model was significant, F(2,136) = 5.368, *p* = 0.002, R^2^ = 0.108.

## 4. Discussion

The main objective of this study was to investigate the link between MW content and source and the ability to perform (time-based) PM intentions. In addition to replicating literature [3,4,5], i.e., the high proportion (30–50%) of daily MW, we expected that individuals spend more time thinking about the future rather than the past, highlighting a prospective bias in the MW. Furthermore, as we argued that PM-oriented thoughts MW episodes would be more deliberate, we expected that engaging in a deliberate form of PM-oriented MW would lead to better PM performance compared to any other spontaneous WM content. To resume, we also wanted to study whether the tendency towards voluntary or spontaneous MW, or the content of MW (past/future), or the interaction between these characteristics predicted the execution of prospective intentions.

### 4.1. Validation of the Mind Wandering’s Experimental Protocol

Coherently with previous findings [4], we observe a strong propensity to perform MW (39,6%) in daily life, thus confirming the prevalence of this human cognitive phenomenon. Additionally, do episodes of MW represent a significant proportion of daily waking time and seem to occur mostly in the context of work (15,1%), during which boredom and external distractions are common. In contrast, as Killingsworth and Gilbert [4] have shown, few or no episodes of MW are experienced during the physical act of love (0,11%). In addition to replicating these observations, our results highlight the presence of a prospective bias in the content of the MW (significantly higher rate of planning-oriented thoughts). Our results are also in line with the literature [62,66,75] and seem to point to a potential function of mind wandering. This could explain why future-oriented thoughts are so common in everyday life [76,77,78]. Actually, it seems unlikely that such a prominent phenomenon of our mental life serves no purpose.

### 4.2. Validation of the Prospective Memory’s Experimental Protocol

Metacognition is a factor strongly related to memory abilities, specifically with PM as it requires us to reflect on our intentions while they are being formed as well as when they are retrieved [79,80]. It is becoming increasingly evident that the metamemory capacities of individuals can modulate the nature of the processes recruited in PM functioning [81,82]. The nature of the processes engaged would thus be defined by our estimation of our attentional and memory capacities and by the difficulty we attribute to the task. It is therefore expected to observe links between metamemory and prospective memory in the same study measuring these two aspects. This would, in a way, support the methodology used. As anticipated, we report a positive relationship between the metamemory’s aptitude, as measured subjectively by the MPMI_s questionnaire [68] and our objective measure of prospective memory. This observation tends to confirm the reliability of our prospective memory task.

### 4.3. Investigating the Link between MW and MP

The existence of the prospective bias within the MW content is therefore confirmed, but its role and origin still raise questions. Contrary to what the literature would lead us to believe our results show that prospective thoughts occur more voluntarily than spontaneously. Indeed, Cole [83] hypothesized that the majority of future thoughts in MW were spontaneous and “pre-fabricated”. Because spontaneous thoughts oriented towards the future would come to consciousness much more quickly than those oriented towards the past, those “ready-made” thoughts would be mainly about future tasks and goals. Thus, each spontaneous future thought would simply be a re-iteration of a previously constructed future event. Furthermore, Stawarczyk et al. [74] theorized that future-oriented MW episodes involved more inner speech, and would be de facto more personally relevant and more realistic or concrete than visual imagery contained in past-oriented thoughts, which are less structured. Inner speech would so allow for the future mental representation of intentions through the internal enunciation of future action, to better plan it. Lastly, in their review, Kvavilashvili and Rummel [37] agree wholeheartedly with this view, by concluding that future-oriented thinking is largely spontaneous, focused on the near future and concerned with concrete projects rather than abstract projections. Nevertheless, thinking about the upcoming prospective memory task in the retention interval could be also deliberate, notably when mentally revising one’s plans for a day [37].

If the MW prospective bias seems to check all the boxes to be a determining factor in the successful realization of an intention, it does not predict prospective memory performance in our study. Quite the opposite, we reported that the mind wandering trait predicts negatively the PM correct ratio. This is not really surprising considering that MW could have a negative effect on PM performance, particularly if it occurs during the encoding or recall phases [29]. Indeed, if during the encoding and planning of future intentions, our mind wanders, the quality of encoding will be strongly altered and will therefore lead to poorer prospective recall performance. Similarly, when an episode of MW occurs during the period in which we should be confronted with various prospective cues, normally allowing us to correctly retrieve the intention in memory, attention is then decoupled from the environment and we cannot be challenged by the salience of the prospective cues, which would in fine go unnoticed [53,54]. Nevertheless, our results seem to rather show a link between past-oriented thoughts and the execution of a prospective intention. This might make sense if you consider that if the MW appears during the intention retention period, after the encoding phase and prior to the recall phase, there would be a potential improvement in prospective memory capacities. Some studies tend to show that MW could contribute to improving memory encoding [31,32,84], and even memory consolidation [33,34]. The recall of a memory leads to a re-encoding of the event, promoting its long-term consolidation. Thus, during a phase of retention of prospective information following prospective encoding, MW would allow for consolidation of the memory of these intentions, by recalling them to mind regularly [85]. This idea is all the more valid since, if we take up the distinction between voluntary and spontaneous MW, i.e., one based on mental imagery and the other on internal speech. Mental imagery in PM, which consists of imagining, at the time of encoding the intention, the context of the action, as well as its actual realization by borrowing a personal perspective, could facilitate the retrieval of intentions in PM. This mechanism of projection into the future deployed at the time of encoding, or re-encoding during the consolidation period, would have a beneficial effect on the subsequent recall of intentions by facilitating the identification and automatic processing of prospective cues [86]. This technique has been shown to improve recall of event-based and time-based intentions [87]. Thus, it is likely that the reactivation or reinforcement of the representation of the intention through the MW in the retention phase would be at the origin of the relationship between past-oriented thoughts and the realization of prospective intentions.

The beneficial intervention of MW during a retention period is also found in the literature, particularly in the theoretical field of creativity. Indeed, some studies seem to point to the possibility that MW (both the state and the trait) improves divergent thinking abilities, as it would allow, through the free association of thoughts, to generate new ideas or solve problems [88,89,90,91]. Thus, the emergence of MW episodes during the incubation phase of a problem would improve its success [92] and allow for greater flexibility in a divergent thinking task [93]. However, this potential benefic aspect does not appear to be systematic [94,95] and could depend on the distance between the establishment of a problem and its resolution: a short distance would mobilize more controlled processes, making the influence of mind wandering harmful, while a longer distance would rely mainly on associative, more automatic processes. It would be mainly in this second case that the beneficial effect of MW would be manifested. Even if the results are relatively heterogeneous, these studies on creativity suggest that the content of mind wandering can be used to create associations with other information, suggesting better processing and retention of this information.

When mind wandering is totally disconnected from what the participant is doing at the moment, the impact of mind wandering will be analogous to a divided attention situation and will negatively impact the processing of environmental stimuli (PM encoding and retrieval). However, when the content of the mind wandering is linked more or less directly with certain external stimuli, it may favor their processing, generating spontaneous associations. If we apply this pattern of results to encoding in episodic memory, then we can predict that, in the majority of cases, mind wandering will have a negative impact on encoding. Nonetheless, in certain specific cases, where the participant experiences thoughts related to the stimuli, this associative processing will probably allow for better encoding them, notably during the PM retention period. In conclusion, while the use of future-oriented thoughts will obviously improve the planning of an intention, it may well be that episodes of MW during the retention period also allow participants to re-encode intentions. This assumption makes sense with the literature, in that past-oriented thoughts would be more mental imagery than inner speech, in contrast to future-oriented thinking [74]. If it is just the inner speech that would be the source of the beneficial impact of prospective bias, notably via the involvement of spontaneous mental rehearsal of an action on its future execution [63,65,96], the beneficial effect of using this mental rehearsal intention technique could be due that we use our episodic memory to imagine future scenarios [97]. We hypothesize that rather than repeating the intention inwardly, in a literal way, we use mental imagery to visually recall the source of the encoding, or imagine ourselves carrying out the intention, associating all the context that surrounds the realization, at the appropriate time, of the prospective action. Past-oriented thoughts would thus serve, in this case, to consolidate an already programmed intention.

Nonetheless, these interpretations remain largely speculative since our two tasks were completely independent. Indeed, we had to distinguish the phases in which we measured MW from those in which we measured the PM. This was performed in order to avoid a bias in our prospective memory task since sending thought probes at the same time as the subjects had to perform the PM task would have likely serve as prospective reminder.

## 5. Conclusions

If we confirm the presence of an important prospective bias, supporting the hypothesis of a memory system strongly committed to the future, we do not find a significant effect of such a bias on the achievement of prospective intentions. On the contrary, we found that past-oriented MW positively predicted PM performances. We propose that this link could be explained by the fact that encoded intentions are re-encoded when reactivated during MW. Thus, MW could enhance intention representation and increase intention retrieval. Although the proposed hypotheses to explain these findings are largely speculative, they are easily testable in future studies. For instance, we could explore the impact of an induced MW condition on PM performance. For example, it would be possible to compare the effect of MW induction after the encoding of prospective intentions to a control condition (with MW removed) on PM performance. For the experimental MW induction, research has already suggested that manipulations of task difficulty (cognitive load) could differentially affect rates of intentional and unintentional MW [55] and this kind of manipulation could have opposing effects on intentional and unintentional MW: whereas participants reported more intentional MW in an easy task (low cognitive load) than in a difficult task (high cognitive load), they reported more unintentional MW in a difficult task than in an easy task [98]. Our results would have been strengthened by using a standardized PM assessment. Nevertheless, the online recruitment on smartphones hardly permits the administration of experimental tasks. This problem unfortunately concerns all PM studies conducted in real-life contexts and it seems that this issue has not yet been solved. Faced with this Cornelian choice, stuck between a lack of ecological validity of PM lab-based task and the lack of experimental control of PM real-life tasks, virtual reality could represent a good compromise to assess PM, bypassing the biases of classical evaluations. By placing subjects in a multitude of naturalistic situations for which they can recall a wide variety of intentions, it allows us to simulate the complexity of the activities of daily life and while maintaining experimental rigor, to obtain a measurement that is both sensitive, complete, and specific to the functioning of the PM and its various components, that is difficult to uphold in real-life PM tasks. We think that such a methodology deserves further attention that future studies should try to definitively settle the question of the benefic role of the prospective bias in the achievement of prospective intentions.

In conclusion, our study is inspiring on the inextricable and surprising link between MW and PM. Firstly, future-oriented thoughts are more voluntary than we think, and secondly, the projection of the MW into the past might play a more important role in the potential function of MW on PM. Additional research is expected to explore the subtle nuances of the MW content. Lastly, the use of other methods (ESM) to measure MW and PM is of interest for the study and understanding of PM outside the laboratory in everyday life.

## Figures and Tables

**Table 1:** Mind wandering’s probes: Descriptive Statistics on 226 participants.

	*TOTAL*	*FOCUS*	*MIND* *WANDERING*
*Sent Probes*	*5424*	*—*	*—*
*Received Probes*	*4465*	*2700*	*1765*
*Average Probes Answered per participants* *(24 thought probes possible)*	*19.75 ± 3.63* *Min–12* *Max–24*	*11.95 ± 4.06*	*7.81 ± 3.67*

**Table 2:** Mind wandering’s probes: Survey questions and their responses and coding.

*Q1—What were you doing just before you got the message?*	Reading, Walking, Houseworking, etc… (23 responses here)
*Q2—Are you…*	*At home, At work, Other*
*Q3—Are you…*	*Alone, With another person, In a group*
*Q4—Were you thinking of anything other than what you were doing?*	*Yes, No*
*Q5—What were you thinking exactly?* ***conditioned on the answer “Yes” to Q4, otherwise end of survey***	- I *was thinking about the future (next holiday, life goals, etc.)—**Episodic Future Thinking—EFT*** - *I was thinking about something specific I need to remember to do in the future (appointment, mail, shopping, etc.)—* **Planning** - *I was thinking of a past event (last holiday, evening with friends, exam or meeting, etc.)—**Episodic Past Thinking—EPT*** - *I was thinking of an imaginary situation (fictive, unreal)—**Imagination*** - *I wasn’t thinking about anything in particular, nor was I focused on a current task—**Nothing***
*Q6—Did this thought arise while…* ***conditioned on the answer “Planning” to Q5, otherwise end of survey***	*You just planned this intention/You had already planned this intention*
*Q7—How did this thought come to your mind?* ***conditioned on the answer “Yes” to Q4***	- *Something in my environment caught my attention and made me think about it—**External*** - *I thought about it as my mind wandered despite myself—**Voluntary*** - *Nothing special reminded me of it, I just thought of it spontaneously—**Spontaneous***

**Table 3:** Prospective memory survey: questions and responses.

*Q1—How often have you thought about doing this task?*	Never/Rarely/Sometimes/Often/Very often
*Q2—Around what time did you think about it for the first time?*	*In the awakening/6 a.m./7 a.m./8 a.m./9 a.m./10 a.m./11 a.m./12 p.m./1 p.m./2 p.m./3 p.m./Just before*
*Q3—Around what time did you think about it for the last time?*	*In the awakening/6 a.m./7 a.m./8 a.m./9 a.m./10 a.m./11 a.m./12 p.m./1 p.m./2 p.m./3 p.m./Just before*
*Q4—How did you remember that you were to complete this task?*	*Something in my environment caught my attention and made me think about it* *I thought about it as my mind wandered despite myself * *Nothing special reminded me of it, I just thought of it spontaneously*
*Q5—Has performing this task become automatic?*	*Yes/No*

**Table 4:** Mind wandering’s probes: Descriptive statistics of ongoing activities during thought probes.

*Activity*	*Frequency*
*Making love*	*0.11%*
*Meditate*	*0.23%*
*Play*	*0.62%*
*Radio news*	*0.74%*
*Sport*	*0.96%*
*Read*	*0.96%*
*Children*	*1.59%*
*Shopping*	*1.87%*
*Walk*	*3.06%*
*Relaxation*	*3.17%*
*Housework*	*3.34%*
*Other*	*3.91%*
*Cooking*	*4.76%*
*Home computer*	*5.27%*
*Talk*	*5.33%*
*Sleep*	*5.84%*
*Watching TV*	*5.84%*
*Ablution*	*7.31%*
*Scrolling phone*	*7.42%*
*Eat*	*8.95%*
*Commuting travel*	*12.92%*
*Work*	*15.81%*

## Data Availability

The data that support the findings of this study are available from the corresponding author upon request.

## Supplementary Materials

The following supporting information can be downloaded at: https://www.mdpi.com/article/10.3390/brainsci12091139/s1, S1: Correlation_Matrix; S2: Interaction-Post_Hoc.
