# Supplementary material for: Where Is My Mind…? The Link between Mind Wandering and Prospective Memory
Source: Brain Sci. 2022 Aug 26;12(9):1139. doi: 10.3390/brainsci12091139 (PMC9497275; doi:10.3390/brainsci12091139)
Supplement: Supplementary file 1 [file brainsci-12-01139-s001.zip › Supplementary_Materials/S1-Correlation_Matrix.html]

JASP 


# Results

## Repeated Measures ANOVA

| Within Subjects Effects | | | | | | | | | | | | | | | |
| --- | --- | --- | --- | --- | --- | --- | --- | --- | --- | --- | --- | --- | --- | --- | --- |
| Cases | | Sum of Squares | | df | | Mean Square | | F | | p | | η² | | η² p | |
| Source MW |  | 0.015 |  | 2 |  | 0.007 |  | 2.735 |  | 0.066 |  | 0.003 |  | 0.012 |  |
| Residuals |  | 1.203 |  | 450 |  | 0.003 |  |  |  |  |  |  |  |  |  |
| Orientation |  | 0.918 |  | 3 |  | 0.306 |  | 189.661 |  | < .001 |  | 0.172 |  | 0.457 |  |
| Residuals |  | 1.089 |  | 675 |  | 0.002 |  |  |  |  |  |  |  |  |  |
| Source MW ✻ Orientation |  | 0.057 |  | 6 |  | 0.009 |  | 6.267 |  | < .001 |  | 0.011 |  | 0.027 |  |
| Residuals |  | 2.046 |  | 1350 |  | 0.002 |  |  |  |  |  |  |  |  |  |
|  | | | | | | | | | | | | | | | |
|  |  |  |  |  |  |  |  |  |  |  |  |  |  |  |  |
| --- | --- | --- | --- | --- | --- | --- | --- | --- | --- | --- | --- | --- | --- | --- | --- |
| *Note.*  Type III Sum of Squares | | | | | | | | | | | | | | | |

| Between Subjects Effects | | | | | | | | | | | |
| --- | --- | --- | --- | --- | --- | --- | --- | --- | --- | --- | --- |
| Cases | | Sum of Squares | | df | | Mean Square | | F | | p | |
| Residuals |  | 0.551 |  | 225 |  | 0.002 |  |  |  |  |  |
|  | | | | | | | | | | | |
|  |  |  |  |  |  |  |  |  |  |  |  |
| --- | --- | --- | --- | --- | --- | --- | --- | --- | --- | --- | --- |
| *Note.*  Type III Sum of Squares | | | | | | | | | | | |

### Descriptives

| Descriptives | | | | | | | | | |
| --- | --- | --- | --- | --- | --- | --- | --- | --- | --- |
| Source MW | | Orientation | | Mean | | SD | | N | |
| External |  | Imagination |  | 0.013 |  | 0.026 |  | 226 |  |
|  |  | Past |  | 0.013 |  | 0.026 |  | 226 |  |
|  |  | EFT |  | 0.024 |  | 0.039 |  | 226 |  |
|  |  | Planning |  | 0.051 |  | 0.065 |  | 226 |  |
| Spontaneous |  | Imagination |  | 0.017 |  | 0.031 |  | 226 |  |
|  |  | Past |  | 0.017 |  | 0.031 |  | 226 |  |
|  |  | EFT |  | 0.033 |  | 0.046 |  | 226 |  |
|  |  | Planning |  | 0.055 |  | 0.060 |  | 226 |  |
| Voluntary |  | Imagination |  | 0.008 |  | 0.023 |  | 226 |  |
|  |  | Past |  | 0.008 |  | 0.023 |  | 226 |  |
|  |  | EFT |  | 0.020 |  | 0.038 |  | 226 |  |
|  |  | Planning |  | 0.068 |  | 0.067 |  | 226 |  |
|  | | | | | | | | | |

#### Descriptives plots

#####

### Marginal Means

| Marginal Means - Orientation | | | | | | | | | |
| --- | --- | --- | --- | --- | --- | --- | --- | --- | --- |
|  | | | | 95% CI for Mean Difference | | | |  | |
| Orientation | | Marginal Mean | | Lower | | Upper | | SE | |
| EFT |  | 0.026 |  | 0.023 |  | 0.029 |  | 0.002 |  |
| Planning |  | 0.058 |  | 0.053 |  | 0.063 |  | 0.002 |  |
| Past |  | 0.013 |  | 0.011 |  | 0.015 |  | 0.001 |  |
| Imagination |  | 0.013 |  | 0.011 |  | 0.015 |  | 0.001 |  |
|  | | | | | | | | | |

### Post Hoc Tests

| Post Hoc Comparisons - Source MW | | | | | | | | | | | | | | | |
| --- | --- | --- | --- | --- | --- | --- | --- | --- | --- | --- | --- | --- | --- | --- | --- |
|  | | | | | | 95% CI for Mean Difference | | | |  | | | | | |
|  | |  | | Mean Difference | | Lower | | Upper | | SE | | t | | p bonf | |
| Spontaneous |  | External |  | 0.005 |  | -5.368e -4 |  | 0.011 |  | 0.002 |  | 2.182 |  | 0.089 |  |
|  |  | Voluntary |  | 0.004 |  | -0.001 |  | 0.010 |  | 0.002 |  | 1.819 |  | 0.209 |  |
| External |  | Voluntary |  | -8.823e -4 |  | -0.007 |  | 0.005 |  | 0.002 |  | -0.363 |  | 1.000 |  |
|  | | | | | | | | | | | | | | | |
|  |  |  |  |  |  |  |  |  |  |  |  |  |  |  |  |
| --- | --- | --- | --- | --- | --- | --- | --- | --- | --- | --- | --- | --- | --- | --- | --- |
| *Note.*  P-value and confidence intervals adjusted for comparing a family of 3 estimates (confidence intervals corrected using the bonferroni method). | | | | | | | | | | | | | | | |
| *Note.*  Results are averaged over the levels of: Orientation | | | | | | | | | | | | | | | |

## Linear Regression

| Model Summary - Correct\_ratio | | | | | | | | | |
| --- | --- | --- | --- | --- | --- | --- | --- | --- | --- |
| Model | | R | | R² | | Adjusted R² | | RMSE | |
| 1 |  | 0.372 |  | 0.139 |  | 0.070 |  | 0.280 |  |
| 2 |  | 0.366 |  | 0.134 |  | 0.073 |  | 0.279 |  |
| 3 |  | 0.363 |  | 0.132 |  | 0.078 |  | 0.278 |  |
| 4 |  | 0.358 |  | 0.128 |  | 0.081 |  | 0.278 |  |
| 5 |  | 0.353 |  | 0.125 |  | 0.084 |  | 0.277 |  |
| 6 |  | 0.347 |  | 0.120 |  | 0.087 |  | 0.277 |  |
| 7 |  | 0.338 |  | 0.114 |  | 0.087 |  | 0.277 |  |
| 8 |  | 0.323 |  | 0.104 |  | 0.084 |  | 0.277 |  |
|  | | | | | | | | | |

| ANOVA | | | | | | | | | | | | | |
| --- | --- | --- | --- | --- | --- | --- | --- | --- | --- | --- | --- | --- | --- |
| Model | |  | | Sum of Squares | | df | | Mean Square | | F | | p | |
| 1 |  | Regression |  | 1.585 |  | 10 |  | 0.158 |  | 2.028 |  | 0.036 |  |
|  |  | Residual |  | 9.849 |  | 126 |  | 0.078 |  |  |  |  |  |
|  |  | Total |  | 11.434 |  | 136 |  |  |  |  |  |  |  |
| 2 |  | Regression |  | 1.534 |  | 9 |  | 0.170 |  | 2.186 |  | 0.027 |  |
|  |  | Residual |  | 9.901 |  | 127 |  | 0.078 |  |  |  |  |  |
|  |  | Total |  | 11.434 |  | 136 |  |  |  |  |  |  |  |
| 3 |  | Regression |  | 1.511 |  | 8 |  | 0.189 |  | 2.436 |  | 0.017 |  |
|  |  | Residual |  | 9.924 |  | 128 |  | 0.078 |  |  |  |  |  |
|  |  | Total |  | 11.434 |  | 136 |  |  |  |  |  |  |  |
| 4 |  | Regression |  | 1.466 |  | 7 |  | 0.209 |  | 2.709 |  | 0.012 |  |
|  |  | Residual |  | 9.969 |  | 129 |  | 0.077 |  |  |  |  |  |
|  |  | Total |  | 11.434 |  | 136 |  |  |  |  |  |  |  |
| 5 |  | Regression |  | 1.428 |  | 6 |  | 0.238 |  | 3.091 |  | 0.007 |  |
|  |  | Residual |  | 10.007 |  | 130 |  | 0.077 |  |  |  |  |  |
|  |  | Total |  | 11.434 |  | 136 |  |  |  |  |  |  |  |
| 6 |  | Regression |  | 1.375 |  | 5 |  | 0.275 |  | 3.581 |  | 0.005 |  |
|  |  | Residual |  | 10.059 |  | 131 |  | 0.077 |  |  |  |  |  |
|  |  | Total |  | 11.434 |  | 136 |  |  |  |  |  |  |  |
| 7 |  | Regression |  | 1.306 |  | 4 |  | 0.327 |  | 4.257 |  | 0.003 |  |
|  |  | Residual |  | 10.128 |  | 132 |  | 0.077 |  |  |  |  |  |
|  |  | Total |  | 11.434 |  | 136 |  |  |  |  |  |  |  |
| 8 |  | Regression |  | 1.193 |  | 3 |  | 0.398 |  | 5.162 |  | 0.002 |  |
|  |  | Residual |  | 10.242 |  | 133 |  | 0.077 |  |  |  |  |  |
|  |  | Total |  | 11.434 |  | 136 |  |  |  |  |  |  |  |
|  | | | | | | | | | | | | | |

| Coefficients | | | | | | | | | | | | | | | | | |
| --- | --- | --- | --- | --- | --- | --- | --- | --- | --- | --- | --- | --- | --- | --- | --- | --- | --- |
|  | | | | | | | | | | | | | | 95% CI | | | |
| Model | |  | | Unstandardized | | Standard Error | | Standardized | | t | | p | | Lower | | Upper | |
| 1 |  | (Intercept) |  | -0.128 |  | 0.285 |  |  |  | -0.449 |  | 0.654 |  | -0.691 |  | 0.436 |  |
|  |  | Total\_MWQ |  | -0.013 |  | 0.007 |  | -0.169 |  | -1.811 |  | 0.073 |  | -0.028 |  | 0.001 |  |
|  |  | mw\_past\_MW |  | 0.348 |  | 0.227 |  | 0.131 |  | 1.530 |  | 0.128 |  | -0.102 |  | 0.797 |  |
|  |  | Expert |  | -0.051 |  | 0.063 |  | -0.078 |  | -0.809 |  | 0.420 |  | -0.177 |  | 0.074 |  |
|  |  | Meta\_Aptitude |  | 0.005 |  | 0.004 |  | 0.127 |  | 1.330 |  | 0.186 |  | -0.002 |  | 0.013 |  |
|  |  | mw\_freq\_MW |  | 0.144 |  | 0.156 |  | 0.081 |  | 0.921 |  | 0.359 |  | -0.165 |  | 0.453 |  |
|  |  | PHQ4\_Depression |  | 0.021 |  | 0.025 |  | 0.096 |  | 0.828 |  | 0.409 |  | -0.029 |  | 0.070 |  |
|  |  | PHQ4\_Anxiety |  | -0.026 |  | 0.023 |  | -0.132 |  | -1.138 |  | 0.257 |  | -0.071 |  | 0.019 |  |
|  |  | Meta\_Internal |  | 0.008 |  | 0.005 |  | 0.159 |  | 1.662 |  | 0.099 |  | -0.002 |  | 0.019 |  |
|  |  | Meta\_External |  | 0.005 |  | 0.005 |  | 0.099 |  | 1.110 |  | 0.269 |  | -0.004 |  | 0.015 |  |
|  |  | Age |  | 0.002 |  | 0.002 |  | 0.081 |  | 0.826 |  | 0.410 |  | -0.003 |  | 0.006 |  |
| 2 |  | (Intercept) |  | -0.090 |  | 0.280 |  |  |  | -0.320 |  | 0.750 |  | -0.645 |  | 0.465 |  |
|  |  | Total\_MWQ |  | -0.013 |  | 0.007 |  | -0.162 |  | -1.738 |  | 0.085 |  | -0.027 |  | 0.002 |  |
|  |  | mw\_past\_MW |  | 0.364 |  | 0.226 |  | 0.137 |  | 1.613 |  | 0.109 |  | -0.083 |  | 0.811 |  |
|  |  | Meta\_Aptitude |  | 0.005 |  | 0.004 |  | 0.120 |  | 1.270 |  | 0.206 |  | -0.003 |  | 0.012 |  |
|  |  | mw\_freq\_MW |  | 0.139 |  | 0.156 |  | 0.078 |  | 0.893 |  | 0.373 |  | -0.169 |  | 0.448 |  |
|  |  | PHQ4\_Depression |  | 0.019 |  | 0.025 |  | 0.090 |  | 0.783 |  | 0.435 |  | -0.030 |  | 0.069 |  |
|  |  | PHQ4\_Anxiety |  | -0.023 |  | 0.023 |  | -0.118 |  | -1.032 |  | 0.304 |  | -0.068 |  | 0.021 |  |
|  |  | Meta\_Internal |  | 0.008 |  | 0.005 |  | 0.155 |  | 1.628 |  | 0.106 |  | -0.002 |  | 0.018 |  |
|  |  | Meta\_External |  | 0.004 |  | 0.005 |  | 0.081 |  | 0.939 |  | 0.350 |  | -0.005 |  | 0.014 |  |
|  |  | Age |  | 0.001 |  | 0.002 |  | 0.048 |  | 0.544 |  | 0.587 |  | -0.003 |  | 0.005 |  |
| 3 |  | (Intercept) |  | -0.017 |  | 0.246 |  |  |  | -0.071 |  | 0.943 |  | -0.505 |  | 0.470 |  |
|  |  | Total\_MWQ |  | -0.014 |  | 0.007 |  | -0.173 |  | -1.922 |  | 0.057 |  | -0.028 |  | 4.077e -4 |  |
|  |  | mw\_past\_MW |  | 0.379 |  | 0.224 |  | 0.143 |  | 1.692 |  | 0.093 |  | -0.064 |  | 0.821 |  |
|  |  | Meta\_Aptitude |  | 0.005 |  | 0.004 |  | 0.123 |  | 1.300 |  | 0.196 |  | -0.003 |  | 0.012 |  |
|  |  | mw\_freq\_MW |  | 0.127 |  | 0.154 |  | 0.071 |  | 0.827 |  | 0.410 |  | -0.177 |  | 0.432 |  |
|  |  | PHQ4\_Depression |  | 0.019 |  | 0.025 |  | 0.088 |  | 0.764 |  | 0.447 |  | -0.030 |  | 0.068 |  |
|  |  | PHQ4\_Anxiety |  | -0.023 |  | 0.022 |  | -0.118 |  | -1.030 |  | 0.305 |  | -0.068 |  | 0.021 |  |
|  |  | Meta\_Internal |  | 0.008 |  | 0.005 |  | 0.151 |  | 1.595 |  | 0.113 |  | -0.002 |  | 0.018 |  |
|  |  | Meta\_External |  | 0.004 |  | 0.005 |  | 0.078 |  | 0.904 |  | 0.368 |  | -0.005 |  | 0.013 |  |
| 4 |  | (Intercept) |  | -0.009 |  | 0.246 |  |  |  | -0.038 |  | 0.970 |  | -0.495 |  | 0.477 |  |
|  |  | Total\_MWQ |  | -0.013 |  | 0.007 |  | -0.164 |  | -1.837 |  | 0.068 |  | -0.027 |  | 9.986e -4 |  |
|  |  | mw\_past\_MW |  | 0.381 |  | 0.223 |  | 0.144 |  | 1.705 |  | 0.091 |  | -0.061 |  | 0.823 |  |
|  |  | Meta\_Aptitude |  | 0.005 |  | 0.004 |  | 0.130 |  | 1.388 |  | 0.168 |  | -0.002 |  | 0.013 |  |
|  |  | mw\_freq\_MW |  | 0.128 |  | 0.154 |  | 0.072 |  | 0.836 |  | 0.405 |  | -0.176 |  | 0.432 |  |
|  |  | PHQ4\_Anxiety |  | -0.012 |  | 0.017 |  | -0.060 |  | -0.701 |  | 0.485 |  | -0.045 |  | 0.021 |  |
|  |  | Meta\_Internal |  | 0.007 |  | 0.005 |  | 0.131 |  | 1.443 |  | 0.151 |  | -0.003 |  | 0.017 |  |
|  |  | Meta\_External |  | 0.005 |  | 0.005 |  | 0.088 |  | 1.036 |  | 0.302 |  | -0.004 |  | 0.014 |  |
| 5 |  | (Intercept) |  | -0.029 |  | 0.244 |  |  |  | -0.121 |  | 0.904 |  | -0.511 |  | 0.452 |  |
|  |  | Total\_MWQ |  | -0.014 |  | 0.007 |  | -0.172 |  | -1.950 |  | 0.053 |  | -0.028 |  | 1.977e -4 |  |
|  |  | mw\_past\_MW |  | 0.393 |  | 0.222 |  | 0.148 |  | 1.767 |  | 0.080 |  | -0.047 |  | 0.832 |  |
|  |  | Meta\_Aptitude |  | 0.005 |  | 0.004 |  | 0.133 |  | 1.428 |  | 0.156 |  | -0.002 |  | 0.013 |  |
|  |  | mw\_freq\_MW |  | 0.127 |  | 0.153 |  | 0.071 |  | 0.827 |  | 0.410 |  | -0.177 |  | 0.430 |  |
|  |  | Meta\_Internal |  | 0.006 |  | 0.005 |  | 0.121 |  | 1.350 |  | 0.179 |  | -0.003 |  | 0.016 |  |
|  |  | Meta\_External |  | 0.005 |  | 0.005 |  | 0.084 |  | 0.996 |  | 0.321 |  | -0.005 |  | 0.014 |  |
| 6 |  | (Intercept) |  | 0.012 |  | 0.238 |  |  |  | 0.051 |  | 0.959 |  | -0.459 |  | 0.483 |  |
|  |  | Total\_MWQ |  | -0.013 |  | 0.007 |  | -0.158 |  | -1.830 |  | 0.069 |  | -0.026 |  | 0.001 |  |
|  |  | mw\_past\_MW |  | 0.425 |  | 0.219 |  | 0.160 |  | 1.943 |  | 0.054 |  | -0.008 |  | 0.857 |  |
|  |  | Meta\_Aptitude |  | 0.005 |  | 0.004 |  | 0.123 |  | 1.332 |  | 0.185 |  | -0.002 |  | 0.012 |  |
|  |  | Meta\_Internal |  | 0.007 |  | 0.005 |  | 0.128 |  | 1.431 |  | 0.155 |  | -0.003 |  | 0.016 |  |
|  |  | Meta\_External |  | 0.004 |  | 0.005 |  | 0.080 |  | 0.945 |  | 0.347 |  | -0.005 |  | 0.013 |  |
| 7 |  | (Intercept) |  | 0.124 |  | 0.206 |  |  |  | 0.602 |  | 0.548 |  | -0.284 |  | 0.532 |  |
|  |  | Total\_MWQ |  | -0.012 |  | 0.007 |  | -0.151 |  | -1.756 |  | 0.081 |  | -0.026 |  | 0.002 |  |
|  |  | mw\_past\_MW |  | 0.427 |  | 0.218 |  | 0.161 |  | 1.957 |  | 0.052 |  | -0.005 |  | 0.860 |  |
|  |  | Meta\_Aptitude |  | 0.004 |  | 0.004 |  | 0.112 |  | 1.218 |  | 0.225 |  | -0.003 |  | 0.012 |  |
|  |  | Meta\_Internal |  | 0.008 |  | 0.005 |  | 0.143 |  | 1.638 |  | 0.104 |  | -0.002 |  | 0.017 |  |
| 8 |  | (Intercept) |  | 0.267 |  | 0.170 |  |  |  | 1.571 |  | 0.118 |  | -0.069 |  | 0.603 |  |
|  |  | Total\_MWQ |  | -0.015 |  | 0.007 |  | -0.183 |  | -2.226 |  | 0.028 |  | -0.027 |  | -0.002 |  |
|  |  | mw\_past\_MW |  | 0.440 |  | 0.219 |  | 0.166 |  | 2.012 |  | 0.046 |  | 0.007 |  | 0.872 |  |
|  |  | Meta\_Internal |  | 0.010 |  | 0.004 |  | 0.180 |  | 2.190 |  | 0.030 |  | 9.296e -4 |  | 0.018 |  |
|  | | | | | | | | | | | | | | | | | |

## Copy of Linear Regression

| Model Summary - mw\_freq\_MW | | | | | | | | | |
| --- | --- | --- | --- | --- | --- | --- | --- | --- | --- |
| Model | | R | | R² | | Adjusted R² | | RMSE | |
| 1 |  | 0.260 |  | 0.067 |  | 0.024 |  | 0.161 |  |
| 2 |  | 0.260 |  | 0.067 |  | 0.032 |  | 0.160 |  |
| 3 |  | 0.259 |  | 0.067 |  | 0.039 |  | 0.160 |  |
| 4 |  | 0.259 |  | 0.067 |  | 0.046 |  | 0.159 |  |
| 5 |  | 0.245 |  | 0.060 |  | 0.046 |  | 0.159 |  |
| 6 |  | 0.210 |  | 0.044 |  | 0.037 |  | 0.160 |  |
|  | | | | | | | | | |

| ANOVA | | | | | | | | | | | | | |
| --- | --- | --- | --- | --- | --- | --- | --- | --- | --- | --- | --- | --- | --- |
| Model | |  | | Sum of Squares | | df | | Mean Square | | F | | p | |
| 1 |  | Regression |  | 0.243 |  | 6 |  | 0.040 |  | 1.568 |  | 0.162 |  |
|  |  | Residual |  | 3.358 |  | 130 |  | 0.026 |  |  |  |  |  |
|  |  | Total |  | 3.601 |  | 136 |  |  |  |  |  |  |  |
| 2 |  | Regression |  | 0.243 |  | 5 |  | 0.049 |  | 1.894 |  | 0.100 |  |
|  |  | Residual |  | 3.358 |  | 131 |  | 0.026 |  |  |  |  |  |
|  |  | Total |  | 3.601 |  | 136 |  |  |  |  |  |  |  |
| 3 |  | Regression |  | 0.242 |  | 4 |  | 0.060 |  | 2.373 |  | 0.055 |  |
|  |  | Residual |  | 3.359 |  | 132 |  | 0.025 |  |  |  |  |  |
|  |  | Total |  | 3.601 |  | 136 |  |  |  |  |  |  |  |
| 4 |  | Regression |  | 0.241 |  | 3 |  | 0.080 |  | 3.180 |  | 0.026 |  |
|  |  | Residual |  | 3.360 |  | 133 |  | 0.025 |  |  |  |  |  |
|  |  | Total |  | 3.601 |  | 136 |  |  |  |  |  |  |  |
| 5 |  | Regression |  | 0.216 |  | 2 |  | 0.108 |  | 4.275 |  | 0.016 |  |
|  |  | Residual |  | 3.385 |  | 134 |  | 0.025 |  |  |  |  |  |
|  |  | Total |  | 3.601 |  | 136 |  |  |  |  |  |  |  |
| 6 |  | Regression |  | 0.159 |  | 1 |  | 0.159 |  | 6.239 |  | 0.014 |  |
|  |  | Residual |  | 3.442 |  | 135 |  | 0.025 |  |  |  |  |  |
|  |  | Total |  | 3.601 |  | 136 |  |  |  |  |  |  |  |
|  | | | | | | | | | | | | | |

| Coefficients | | | | | | | | | | | | | | | | | |
| --- | --- | --- | --- | --- | --- | --- | --- | --- | --- | --- | --- | --- | --- | --- | --- | --- | --- |
|  | | | | | | | | | | | | | | 95% CI | | | |
| Model | |  | | Unstandardized | | Standard Error | | Standardized | | t | | p | | Lower | | Upper | |
| 1 |  | (Intercept) |  | 0.422 |  | 0.133 |  |  |  | 3.167 |  | 0.002 |  | 0.158 |  | 0.685 |  |
|  |  | Meta\_Aptitude |  | -0.002 |  | 0.002 |  | -0.088 |  | -0.990 |  | 0.324 |  | -0.006 |  | 0.002 |  |
|  |  | Total\_MWQ |  | 0.007 |  | 0.004 |  | 0.147 |  | 1.555 |  | 0.122 |  | -0.002 |  | 0.015 |  |
|  |  | Expert |  | 0.003 |  | 0.035 |  | 0.008 |  | 0.082 |  | 0.935 |  | -0.066 |  | 0.072 |  |
|  |  | Age |  | -0.002 |  | 0.001 |  | -0.131 |  | -1.366 |  | 0.174 |  | -0.004 |  | 7.677e -4 |  |
|  |  | PHQ4\_Depression |  | -0.004 |  | 0.014 |  | -0.029 |  | -0.257 |  | 0.798 |  | -0.031 |  | 0.024 |  |
|  |  | PHQ4\_Anxiety |  | 0.003 |  | 0.012 |  | 0.025 |  | 0.224 |  | 0.823 |  | -0.022 |  | 0.027 |  |
| 2 |  | (Intercept) |  | 0.421 |  | 0.133 |  |  |  | 3.178 |  | 0.002 |  | 0.159 |  | 0.684 |  |
|  |  | Meta\_Aptitude |  | -0.002 |  | 0.002 |  | -0.088 |  | -0.990 |  | 0.324 |  | -0.006 |  | 0.002 |  |
|  |  | Total\_MWQ |  | 0.007 |  | 0.004 |  | 0.146 |  | 1.559 |  | 0.121 |  | -0.002 |  | 0.015 |  |
|  |  | Age |  | -0.002 |  | 0.001 |  | -0.129 |  | -1.445 |  | 0.151 |  | -0.004 |  | 6.180e -4 |  |
|  |  | PHQ4\_Depression |  | -0.003 |  | 0.014 |  | -0.028 |  | -0.253 |  | 0.801 |  | -0.030 |  | 0.024 |  |
|  |  | PHQ4\_Anxiety |  | 0.003 |  | 0.012 |  | 0.024 |  | 0.216 |  | 0.829 |  | -0.022 |  | 0.027 |  |
| 3 |  | (Intercept) |  | 0.425 |  | 0.131 |  |  |  | 3.244 |  | 0.001 |  | 0.166 |  | 0.684 |  |
|  |  | Meta\_Aptitude |  | -0.002 |  | 0.002 |  | -0.088 |  | -0.993 |  | 0.322 |  | -0.006 |  | 0.002 |  |
|  |  | Total\_MWQ |  | 0.007 |  | 0.004 |  | 0.147 |  | 1.571 |  | 0.119 |  | -0.002 |  | 0.015 |  |
|  |  | Age |  | -0.002 |  | 0.001 |  | -0.129 |  | -1.456 |  | 0.148 |  | -0.004 |  | 6.025e -4 |  |
|  |  | PHQ4\_Depression |  | -0.002 |  | 0.010 |  | -0.013 |  | -0.149 |  | 0.882 |  | -0.022 |  | 0.019 |  |
| 4 |  | (Intercept) |  | 0.421 |  | 0.128 |  |  |  | 3.294 |  | 0.001 |  | 0.168 |  | 0.674 |  |
|  |  | Meta\_Aptitude |  | -0.002 |  | 0.002 |  | -0.087 |  | -0.996 |  | 0.321 |  | -0.006 |  | 0.002 |  |
|  |  | Total\_MWQ |  | 0.006 |  | 0.004 |  | 0.144 |  | 1.576 |  | 0.117 |  | -0.002 |  | 0.014 |  |
|  |  | Age |  | -0.002 |  | 0.001 |  | -0.128 |  | -1.456 |  | 0.148 |  | -0.004 |  | 5.999e -4 |  |
| 5 |  | (Intercept) |  | 0.338 |  | 0.097 |  |  |  | 3.490 |  | < .001 |  | 0.146 |  | 0.530 |  |
|  |  | Total\_MWQ |  | 0.008 |  | 0.004 |  | 0.169 |  | 1.921 |  | 0.057 |  | -2.217e -4 |  | 0.015 |  |
|  |  | Age |  | -0.002 |  | 0.001 |  | -0.132 |  | -1.501 |  | 0.136 |  | -0.004 |  | 5.469e -4 |  |
| 6 |  | (Intercept) |  | 0.240 |  | 0.072 |  |  |  | 3.339 |  | 0.001 |  | 0.098 |  | 0.382 |  |
|  |  | Total\_MWQ |  | 0.009 |  | 0.004 |  | 0.210 |  | 2.498 |  | 0.014 |  | 0.002 |  | 0.017 |  |
|  | | | | | | | | | | | | | | | | | |

## Descriptive Statistics

| Descriptive Statistics | | | |
| --- | --- | --- | --- |
|  | | n\_telephone | |
| Valid |  | 226 |  |
| Missing |  | 0 |  |
| Mean |  | 1.150e +10 |  |
| Std. Deviation |  | 5.705e +10 |  |
| Minimum |  | 4.709e +8 |  |
| Maximum |  | 4.480e +11 |  |
|  | | | |

## Correlation

| Pearson's Correlations | | | | | | | | | | | | | | | | | | | | | | | | | | | | | | | | | | | | | | | | | | | | | | | | | | | | | | | | | | | | | | | | | | | |
| --- | --- | --- | --- | --- | --- | --- | --- | --- | --- | --- | --- | --- | --- | --- | --- | --- | --- | --- | --- | --- | --- | --- | --- | --- | --- | --- | --- | --- | --- | --- | --- | --- | --- | --- | --- | --- | --- | --- | --- | --- | --- | --- | --- | --- | --- | --- | --- | --- | --- | --- | --- | --- | --- | --- | --- | --- | --- | --- | --- | --- | --- | --- | --- | --- | --- | --- | --- |
| Variable | |  | | mw\_freq\_TOT | | mw\_rien\_TOT | | mw\_future\_thinking\_TOT | | mw\_planning\_TOT | | mw\_past\_TOT | | mw\_imagination\_TOT | | mw\_spontaneous\_TOT | | mw\_externe\_TOT | | mw\_voluntary\_TOT | | mw\_spontaneous\_EFT\_TOT | | mw\_externe\_EFT\_TOT | | mw\_voluntary\_EFT\_TOT | | mw\_spontaneous\_MP\_TOT | | mw\_externe\_MP\_TOT | | mw\_voluntary\_MP\_TOT | | mw\_spontaneous\_Past\_Oriented\_TOT | | mw\_externe\_Past\_Oriented\_TOT | | mw\_voluntary\_Past\_Oriented\_TOT | | mw\_spontaneous\_Imagination\_TOT | | mw\_externe\_Imagination\_TOT | | mw\_voluntary\_Imagination\_TOT | | Total\_MWQ | | Correct\_ratio | | Incorrect\_ratio | | Omission\_ratio | | Meta\_Aptitude | | Meta\_Internal | | Meta\_External | | Age | | Expert | | PHQ4\_Depression | | PHQ4\_Anxiety | |
| 1. mw\_freq\_TOT |  | Pearson's r |  | — |  |  |  |  |  |  |  |  |  |  |  |  |  |  |  |  |  |  |  |  |  |  |  |  |  |  |  |  |  |  |  |  |  |  |  |  |  |  |  |  |  |  |  |  |  |  |  |  |  |  |  |  |  |  |  |  |  |  |  |  |  |  |  |
|  |  | p-value |  | — |  |  |  |  |  |  |  |  |  |  |  |  |  |  |  |  |  |  |  |  |  |  |  |  |  |  |  |  |  |  |  |  |  |  |  |  |  |  |  |  |  |  |  |  |  |  |  |  |  |  |  |  |  |  |  |  |  |  |  |  |  |  |  |
|  |  | Upper 95% CI |  | — |  |  |  |  |  |  |  |  |  |  |  |  |  |  |  |  |  |  |  |  |  |  |  |  |  |  |  |  |  |  |  |  |  |  |  |  |  |  |  |  |  |  |  |  |  |  |  |  |  |  |  |  |  |  |  |  |  |  |  |  |  |  |  |
|  |  | Lower 95% CI |  | — |  |  |  |  |  |  |  |  |  |  |  |  |  |  |  |  |  |  |  |  |  |  |  |  |  |  |  |  |  |  |  |  |  |  |  |  |  |  |  |  |  |  |  |  |  |  |  |  |  |  |  |  |  |  |  |  |  |  |  |  |  |  |  |
| 2. mw\_rien\_TOT |  | Pearson's r |  | 0.287 | \*\*\* | — |  |  |  |  |  |  |  |  |  |  |  |  |  |  |  |  |  |  |  |  |  |  |  |  |  |  |  |  |  |  |  |  |  |  |  |  |  |  |  |  |  |  |  |  |  |  |  |  |  |  |  |  |  |  |  |  |  |  |  |  |  |
|  |  | p-value |  | < .001 |  | — |  |  |  |  |  |  |  |  |  |  |  |  |  |  |  |  |  |  |  |  |  |  |  |  |  |  |  |  |  |  |  |  |  |  |  |  |  |  |  |  |  |  |  |  |  |  |  |  |  |  |  |  |  |  |  |  |  |  |  |  |  |
|  |  | Upper 95% CI |  | 0.402 |  | — |  |  |  |  |  |  |  |  |  |  |  |  |  |  |  |  |  |  |  |  |  |  |  |  |  |  |  |  |  |  |  |  |  |  |  |  |  |  |  |  |  |  |  |  |  |  |  |  |  |  |  |  |  |  |  |  |  |  |  |  |  |
|  |  | Lower 95% CI |  | 0.162 |  | — |  |  |  |  |  |  |  |  |  |  |  |  |  |  |  |  |  |  |  |  |  |  |  |  |  |  |  |  |  |  |  |  |  |  |  |  |  |  |  |  |  |  |  |  |  |  |  |  |  |  |  |  |  |  |  |  |  |  |  |  |  |
| 3. mw\_future\_thinking\_TOT |  | Pearson's r |  | 0.436 | \*\*\* | -0.101 |  | — |  |  |  |  |  |  |  |  |  |  |  |  |  |  |  |  |  |  |  |  |  |  |  |  |  |  |  |  |  |  |  |  |  |  |  |  |  |  |  |  |  |  |  |  |  |  |  |  |  |  |  |  |  |  |  |  |  |  |  |
|  |  | p-value |  | < .001 |  | 0.132 |  | — |  |  |  |  |  |  |  |  |  |  |  |  |  |  |  |  |  |  |  |  |  |  |  |  |  |  |  |  |  |  |  |  |  |  |  |  |  |  |  |  |  |  |  |  |  |  |  |  |  |  |  |  |  |  |  |  |  |  |  |
|  |  | Upper 95% CI |  | 0.536 |  | 0.030 |  | — |  |  |  |  |  |  |  |  |  |  |  |  |  |  |  |  |  |  |  |  |  |  |  |  |  |  |  |  |  |  |  |  |  |  |  |  |  |  |  |  |  |  |  |  |  |  |  |  |  |  |  |  |  |  |  |  |  |  |  |
|  |  | Lower 95% CI |  | 0.324 |  | -0.228 |  | — |  |  |  |  |  |  |  |  |  |  |  |  |  |  |  |  |  |  |  |  |  |  |  |  |  |  |  |  |  |  |  |  |  |  |  |  |  |  |  |  |  |  |  |  |  |  |  |  |  |  |  |  |  |  |  |  |  |  |  |
| 4. mw\_planning\_TOT |  | Pearson's r |  | 0.613 | \*\*\* | -0.023 |  | -0.010 |  | — |  |  |  |  |  |  |  |  |  |  |  |  |  |  |  |  |  |  |  |  |  |  |  |  |  |  |  |  |  |  |  |  |  |  |  |  |  |  |  |  |  |  |  |  |  |  |  |  |  |  |  |  |  |  |  |  |  |
|  |  | p-value |  | < .001 |  | 0.731 |  | 0.886 |  | — |  |  |  |  |  |  |  |  |  |  |  |  |  |  |  |  |  |  |  |  |  |  |  |  |  |  |  |  |  |  |  |  |  |  |  |  |  |  |  |  |  |  |  |  |  |  |  |  |  |  |  |  |  |  |  |  |  |
|  |  | Upper 95% CI |  | 0.688 |  | 0.108 |  | 0.121 |  | — |  |  |  |  |  |  |  |  |  |  |  |  |  |  |  |  |  |  |  |  |  |  |  |  |  |  |  |  |  |  |  |  |  |  |  |  |  |  |  |  |  |  |  |  |  |  |  |  |  |  |  |  |  |  |  |  |  |
|  |  | Lower 95% CI |  | 0.524 |  | -0.153 |  | -0.140 |  | — |  |  |  |  |  |  |  |  |  |  |  |  |  |  |  |  |  |  |  |  |  |  |  |  |  |  |  |  |  |  |  |  |  |  |  |  |  |  |  |  |  |  |  |  |  |  |  |  |  |  |  |  |  |  |  |  |  |
| 5. mw\_past\_TOT |  | Pearson's r |  | 0.424 | \*\*\* | -0.059 |  | 0.088 |  | 0.085 |  | — |  |  |  |  |  |  |  |  |  |  |  |  |  |  |  |  |  |  |  |  |  |  |  |  |  |  |  |  |  |  |  |  |  |  |  |  |  |  |  |  |  |  |  |  |  |  |  |  |  |  |  |  |  |  |  |
|  |  | p-value |  | < .001 |  | 0.378 |  | 0.187 |  | 0.203 |  | — |  |  |  |  |  |  |  |  |  |  |  |  |  |  |  |  |  |  |  |  |  |  |  |  |  |  |  |  |  |  |  |  |  |  |  |  |  |  |  |  |  |  |  |  |  |  |  |  |  |  |  |  |  |  |  |
|  |  | Upper 95% CI |  | 0.526 |  | 0.072 |  | 0.216 |  | 0.213 |  | — |  |  |  |  |  |  |  |  |  |  |  |  |  |  |  |  |  |  |  |  |  |  |  |  |  |  |  |  |  |  |  |  |  |  |  |  |  |  |  |  |  |  |  |  |  |  |  |  |  |  |  |  |  |  |  |
|  |  | Lower 95% CI |  | 0.311 |  | -0.188 |  | -0.043 |  | -0.046 |  | — |  |  |  |  |  |  |  |  |  |  |  |  |  |  |  |  |  |  |  |  |  |  |  |  |  |  |  |  |  |  |  |  |  |  |  |  |  |  |  |  |  |  |  |  |  |  |  |  |  |  |  |  |  |  |  |
| 6. mw\_imagination\_TOT |  | Pearson's r |  | 0.394 | \*\*\* | -0.110 |  | 0.050 |  | -0.102 |  | 0.154 | \* | — |  |  |  |  |  |  |  |  |  |  |  |  |  |  |  |  |  |  |  |  |  |  |  |  |  |  |  |  |  |  |  |  |  |  |  |  |  |  |  |  |  |  |  |  |  |  |  |  |  |  |  |  |  |
|  |  | p-value |  | < .001 |  | 0.098 |  | 0.455 |  | 0.128 |  | 0.020 |  | — |  |  |  |  |  |  |  |  |  |  |  |  |  |  |  |  |  |  |  |  |  |  |  |  |  |  |  |  |  |  |  |  |  |  |  |  |  |  |  |  |  |  |  |  |  |  |  |  |  |  |  |  |  |
|  |  | Upper 95% CI |  | 0.499 |  | 0.021 |  | 0.179 |  | 0.029 |  | 0.279 |  | — |  |  |  |  |  |  |  |  |  |  |  |  |  |  |  |  |  |  |  |  |  |  |  |  |  |  |  |  |  |  |  |  |  |  |  |  |  |  |  |  |  |  |  |  |  |  |  |  |  |  |  |  |  |
|  |  | Lower 95% CI |  | 0.278 |  | -0.237 |  | -0.081 |  | -0.229 |  | 0.024 |  | — |  |  |  |  |  |  |  |  |  |  |  |  |  |  |  |  |  |  |  |  |  |  |  |  |  |  |  |  |  |  |  |  |  |  |  |  |  |  |  |  |  |  |  |  |  |  |  |  |  |  |  |  |  |
| 7. mw\_spontaneous\_TOT |  | Pearson's r |  | 0.642 | \*\*\* | -0.035 |  | 0.345 | \*\*\* | 0.394 | \*\*\* | 0.309 | \*\*\* | 0.368 | \*\*\* | — |  |  |  |  |  |  |  |  |  |  |  |  |  |  |  |  |  |  |  |  |  |  |  |  |  |  |  |  |  |  |  |  |  |  |  |  |  |  |  |  |  |  |  |  |  |  |  |  |  |  |  |
|  |  | p-value |  | < .001 |  | 0.596 |  | < .001 |  | < .001 |  | < .001 |  | < .001 |  | — |  |  |  |  |  |  |  |  |  |  |  |  |  |  |  |  |  |  |  |  |  |  |  |  |  |  |  |  |  |  |  |  |  |  |  |  |  |  |  |  |  |  |  |  |  |  |  |  |  |  |  |
|  |  | Upper 95% CI |  | 0.712 |  | 0.096 |  | 0.455 |  | 0.499 |  | 0.423 |  | 0.476 |  | — |  |  |  |  |  |  |  |  |  |  |  |  |  |  |  |  |  |  |  |  |  |  |  |  |  |  |  |  |  |  |  |  |  |  |  |  |  |  |  |  |  |  |  |  |  |  |  |  |  |  |  |
|  |  | Lower 95% CI |  | 0.558 |  | -0.165 |  | 0.225 |  | 0.278 |  | 0.186 |  | 0.249 |  | — |  |  |  |  |  |  |  |  |  |  |  |  |  |  |  |  |  |  |  |  |  |  |  |  |  |  |  |  |  |  |  |  |  |  |  |  |  |  |  |  |  |  |  |  |  |  |  |  |  |  |  |
| 8. mw\_externe\_TOT |  | Pearson's r |  | 0.509 | \*\*\* | -0.076 |  | 0.278 | \*\*\* | 0.330 | \*\*\* | 0.276 | \*\*\* | 0.287 | \*\*\* | 0.064 |  | — |  |  |  |  |  |  |  |  |  |  |  |  |  |  |  |  |  |  |  |  |  |  |  |  |  |  |  |  |  |  |  |  |  |  |  |  |  |  |  |  |  |  |  |  |  |  |  |  |  |
|  |  | p-value |  | < .001 |  | 0.256 |  | < .001 |  | < .001 |  | < .001 |  | < .001 |  | 0.338 |  | — |  |  |  |  |  |  |  |  |  |  |  |  |  |  |  |  |  |  |  |  |  |  |  |  |  |  |  |  |  |  |  |  |  |  |  |  |  |  |  |  |  |  |  |  |  |  |  |  |  |
|  |  | Upper 95% CI |  | 0.600 |  | 0.055 |  | 0.395 |  | 0.441 |  | 0.392 |  | 0.402 |  | 0.193 |  | — |  |  |  |  |  |  |  |  |  |  |  |  |  |  |  |  |  |  |  |  |  |  |  |  |  |  |  |  |  |  |  |  |  |  |  |  |  |  |  |  |  |  |  |  |  |  |  |  |  |
|  |  | Lower 95% CI |  | 0.405 |  | -0.204 |  | 0.153 |  | 0.208 |  | 0.151 |  | 0.162 |  | -0.067 |  | — |  |  |  |  |  |  |  |  |  |  |  |  |  |  |  |  |  |  |  |  |  |  |  |  |  |  |  |  |  |  |  |  |  |  |  |  |  |  |  |  |  |  |  |  |  |  |  |  |  |
| 9. mw\_voluntary\_TOT |  | Pearson's r |  | 0.375 | \*\*\* | -0.116 |  | 0.203 | \*\* | 0.369 | \*\*\* | 0.192 | \*\* | 0.090 |  | -0.020 |  | -0.128 |  | — |  |  |  |  |  |  |  |  |  |  |  |  |  |  |  |  |  |  |  |  |  |  |  |  |  |  |  |  |  |  |  |  |  |  |  |  |  |  |  |  |  |  |  |  |  |  |  |
|  |  | p-value |  | < .001 |  | 0.082 |  | 0.002 |  | < .001 |  | 0.004 |  | 0.176 |  | 0.764 |  | 0.054 |  | — |  |  |  |  |  |  |  |  |  |  |  |  |  |  |  |  |  |  |  |  |  |  |  |  |  |  |  |  |  |  |  |  |  |  |  |  |  |  |  |  |  |  |  |  |  |  |  |
|  |  | Upper 95% CI |  | 0.482 |  | 0.015 |  | 0.325 |  | 0.477 |  | 0.315 |  | 0.218 |  | 0.111 |  | 0.002 |  | — |  |  |  |  |  |  |  |  |  |  |  |  |  |  |  |  |  |  |  |  |  |  |  |  |  |  |  |  |  |  |  |  |  |  |  |  |  |  |  |  |  |  |  |  |  |  |  |
|  |  | Lower 95% CI |  | 0.257 |  | -0.243 |  | 0.075 |  | 0.251 |  | 0.063 |  | -0.041 |  | -0.150 |  | -0.255 |  | — |  |  |  |  |  |  |  |  |  |  |  |  |  |  |  |  |  |  |  |  |  |  |  |  |  |  |  |  |  |  |  |  |  |  |  |  |  |  |  |  |  |  |  |  |  |  |  |
| 10. mw\_spontaneous\_EFT\_TOT |  | Pearson's r |  | 0.339 | \*\*\* | 0.006 |  | 0.646 | \*\*\* | 0.035 |  | 0.037 |  | 0.049 |  | 0.585 | \*\*\* | -0.012 |  | -0.049 |  | — |  |  |  |  |  |  |  |  |  |  |  |  |  |  |  |  |  |  |  |  |  |  |  |  |  |  |  |  |  |  |  |  |  |  |  |  |  |  |  |  |  |  |  |  |  |
|  |  | p-value |  | < .001 |  | 0.931 |  | < .001 |  | 0.600 |  | 0.579 |  | 0.465 |  | < .001 |  | 0.858 |  | 0.460 |  | — |  |  |  |  |  |  |  |  |  |  |  |  |  |  |  |  |  |  |  |  |  |  |  |  |  |  |  |  |  |  |  |  |  |  |  |  |  |  |  |  |  |  |  |  |  |
|  |  | Upper 95% CI |  | 0.450 |  | 0.136 |  | 0.716 |  | 0.165 |  | 0.167 |  | 0.178 |  | 0.665 |  | 0.119 |  | 0.082 |  | — |  |  |  |  |  |  |  |  |  |  |  |  |  |  |  |  |  |  |  |  |  |  |  |  |  |  |  |  |  |  |  |  |  |  |  |  |  |  |  |  |  |  |  |  |  |
|  |  | Lower 95% CI |  | 0.219 |  | -0.125 |  | 0.563 |  | -0.096 |  | -0.094 |  | -0.082 |  | 0.492 |  | -0.142 |  | -0.179 |  | — |  |  |  |  |  |  |  |  |  |  |  |  |  |  |  |  |  |  |  |  |  |  |  |  |  |  |  |  |  |  |  |  |  |  |  |  |  |  |  |  |  |  |  |  |  |
| 11. mw\_externe\_EFT\_TOT |  | Pearson's r |  | 0.256 | \*\*\* | -0.088 |  | 0.560 | \*\*\* | 0.021 |  | 0.043 |  | 0.049 |  | -0.005 |  | 0.545 | \*\*\* | -0.025 |  | -0.010 |  | — |  |  |  |  |  |  |  |  |  |  |  |  |  |  |  |  |  |  |  |  |  |  |  |  |  |  |  |  |  |  |  |  |  |  |  |  |  |  |  |  |  |  |  |
|  |  | p-value |  | < .001 |  | 0.186 |  | < .001 |  | 0.752 |  | 0.520 |  | 0.464 |  | 0.938 |  | < .001 |  | 0.709 |  | 0.887 |  | — |  |  |  |  |  |  |  |  |  |  |  |  |  |  |  |  |  |  |  |  |  |  |  |  |  |  |  |  |  |  |  |  |  |  |  |  |  |  |  |  |  |  |  |
|  |  | Upper 95% CI |  | 0.374 |  | 0.043 |  | 0.644 |  | 0.151 |  | 0.173 |  | 0.178 |  | 0.125 |  | 0.631 |  | 0.106 |  | 0.121 |  | — |  |  |  |  |  |  |  |  |  |  |  |  |  |  |  |  |  |  |  |  |  |  |  |  |  |  |  |  |  |  |  |  |  |  |  |  |  |  |  |  |  |  |  |
|  |  | Lower 95% CI |  | 0.130 |  | -0.216 |  | 0.464 |  | -0.110 |  | -0.088 |  | -0.082 |  | -0.136 |  | 0.447 |  | -0.155 |  | -0.140 |  | — |  |  |  |  |  |  |  |  |  |  |  |  |  |  |  |  |  |  |  |  |  |  |  |  |  |  |  |  |  |  |  |  |  |  |  |  |  |  |  |  |  |  |  |
| 12. mw\_voluntary\_EFT\_TOT |  | Pearson's r |  | 0.167 | \* | -0.111 |  | 0.571 | \*\*\* | -0.084 |  | 0.081 |  | -0.014 |  | -0.042 |  | -0.010 |  | 0.483 | \*\*\* | 0.039 |  | 0.063 |  | — |  |  |  |  |  |  |  |  |  |  |  |  |  |  |  |  |  |  |  |  |  |  |  |  |  |  |  |  |  |  |  |  |  |  |  |  |  |  |  |  |  |
|  |  | p-value |  | 0.012 |  | 0.095 |  | < .001 |  | 0.210 |  | 0.223 |  | 0.837 |  | 0.527 |  | 0.879 |  | < .001 |  | 0.561 |  | 0.347 |  | — |  |  |  |  |  |  |  |  |  |  |  |  |  |  |  |  |  |  |  |  |  |  |  |  |  |  |  |  |  |  |  |  |  |  |  |  |  |  |  |  |  |
|  |  | Upper 95% CI |  | 0.291 |  | 0.019 |  | 0.653 |  | 0.047 |  | 0.210 |  | 0.117 |  | 0.089 |  | 0.120 |  | 0.577 |  | 0.168 |  | 0.192 |  | — |  |  |  |  |  |  |  |  |  |  |  |  |  |  |  |  |  |  |  |  |  |  |  |  |  |  |  |  |  |  |  |  |  |  |  |  |  |  |  |  |  |
|  |  | Lower 95% CI |  | 0.037 |  | -0.238 |  | 0.476 |  | -0.212 |  | -0.050 |  | -0.144 |  | -0.172 |  | -0.140 |  | 0.376 |  | -0.092 |  | -0.068 |  | — |  |  |  |  |  |  |  |  |  |  |  |  |  |  |  |  |  |  |  |  |  |  |  |  |  |  |  |  |  |  |  |  |  |  |  |  |  |  |  |  |  |
| 13. mw\_spontaneous\_MP\_TOT |  | Pearson's r |  | 0.450 | \*\*\* | 0.024 |  | 0.040 |  | 0.606 | \*\*\* | 0.106 |  | 5.843e -5 |  | 0.710 | \*\*\* | 0.007 |  | -0.023 |  | 0.132 | \* | -0.023 |  | -0.061 |  | — |  |  |  |  |  |  |  |  |  |  |  |  |  |  |  |  |  |  |  |  |  |  |  |  |  |  |  |  |  |  |  |  |  |  |  |  |  |  |  |
|  |  | p-value |  | < .001 |  | 0.720 |  | 0.551 |  | < .001 |  | 0.111 |  | 0.999 |  | < .001 |  | 0.915 |  | 0.735 |  | 0.048 |  | 0.736 |  | 0.360 |  | — |  |  |  |  |  |  |  |  |  |  |  |  |  |  |  |  |  |  |  |  |  |  |  |  |  |  |  |  |  |  |  |  |  |  |  |  |  |  |  |
|  |  | Upper 95% CI |  | 0.548 |  | 0.154 |  | 0.169 |  | 0.683 |  | 0.234 |  | 0.131 |  | 0.770 |  | 0.138 |  | 0.108 |  | 0.258 |  | 0.108 |  | 0.070 |  | — |  |  |  |  |  |  |  |  |  |  |  |  |  |  |  |  |  |  |  |  |  |  |  |  |  |  |  |  |  |  |  |  |  |  |  |  |  |  |  |
|  |  | Lower 95% CI |  | 0.339 |  | -0.107 |  | -0.091 |  | 0.517 |  | -0.024 |  | -0.130 |  | 0.639 |  | -0.123 |  | -0.153 |  | 0.001 |  | -0.153 |  | -0.190 |  | — |  |  |  |  |  |  |  |  |  |  |  |  |  |  |  |  |  |  |  |  |  |  |  |  |  |  |  |  |  |  |  |  |  |  |  |  |  |  |  |
| 14. mw\_externe\_MP\_TOT |  | Pearson's r |  | 0.304 | \*\*\* | -0.020 |  | 0.007 |  | 0.527 | \*\*\* | 0.057 |  | -0.110 |  | -4.003e -4 |  | 0.711 | \*\*\* | -0.176 | \*\* | 0.005 |  | 0.095 |  | -0.092 |  | 0.024 |  | — |  |  |  |  |  |  |  |  |  |  |  |  |  |  |  |  |  |  |  |  |  |  |  |  |  |  |  |  |  |  |  |  |  |  |  |  |  |
|  |  | p-value |  | < .001 |  | 0.769 |  | 0.916 |  | < .001 |  | 0.396 |  | 0.100 |  | 0.995 |  | < .001 |  | 0.008 |  | 0.936 |  | 0.155 |  | 0.170 |  | 0.716 |  | — |  |  |  |  |  |  |  |  |  |  |  |  |  |  |  |  |  |  |  |  |  |  |  |  |  |  |  |  |  |  |  |  |  |  |  |  |  |
|  |  | Upper 95% CI |  | 0.418 |  | 0.111 |  | 0.137 |  | 0.616 |  | 0.186 |  | 0.021 |  | 0.130 |  | 0.770 |  | -0.046 |  | 0.136 |  | 0.223 |  | 0.039 |  | 0.154 |  | — |  |  |  |  |  |  |  |  |  |  |  |  |  |  |  |  |  |  |  |  |  |  |  |  |  |  |  |  |  |  |  |  |  |  |  |  |  |
|  |  | Lower 95% CI |  | 0.181 |  | -0.150 |  | -0.124 |  | 0.426 |  | -0.074 |  | -0.237 |  | -0.131 |  | 0.640 |  | -0.299 |  | -0.125 |  | -0.036 |  | -0.219 |  | -0.106 |  | — |  |  |  |  |  |  |  |  |  |  |  |  |  |  |  |  |  |  |  |  |  |  |  |  |  |  |  |  |  |  |  |  |  |  |  |  |  |
| 15. mw\_voluntary\_MP\_TOT |  | Pearson's r |  | 0.297 | \*\*\* | -0.040 |  | -0.058 |  | 0.569 | \*\*\* | -0.013 |  | -0.059 |  | 9.105e -4 |  | -0.158 | \* | 0.792 | \*\*\* | -0.067 |  | -0.037 |  | 0.008 |  | 0.061 |  | -0.133 | \* | — |  |  |  |  |  |  |  |  |  |  |  |  |  |  |  |  |  |  |  |  |  |  |  |  |  |  |  |  |  |  |  |  |  |  |  |
|  |  | p-value |  | < .001 |  | 0.550 |  | 0.382 |  | < .001 |  | 0.851 |  | 0.378 |  | 0.989 |  | 0.017 |  | < .001 |  | 0.314 |  | 0.578 |  | 0.909 |  | 0.364 |  | 0.046 |  | — |  |  |  |  |  |  |  |  |  |  |  |  |  |  |  |  |  |  |  |  |  |  |  |  |  |  |  |  |  |  |  |  |  |  |  |
|  |  | Upper 95% CI |  | 0.411 |  | 0.091 |  | 0.073 |  | 0.651 |  | 0.118 |  | 0.072 |  | 0.131 |  | -0.029 |  | 0.836 |  | 0.064 |  | 0.094 |  | 0.138 |  | 0.190 |  | -0.002 |  | — |  |  |  |  |  |  |  |  |  |  |  |  |  |  |  |  |  |  |  |  |  |  |  |  |  |  |  |  |  |  |  |  |  |  |  |
|  |  | Lower 95% CI |  | 0.173 |  | -0.170 |  | -0.187 |  | 0.474 |  | -0.143 |  | -0.188 |  | -0.130 |  | -0.283 |  | 0.738 |  | -0.196 |  | -0.167 |  | -0.123 |  | -0.070 |  | -0.259 |  | — |  |  |  |  |  |  |  |  |  |  |  |  |  |  |  |  |  |  |  |  |  |  |  |  |  |  |  |  |  |  |  |  |  |  |  |
| 16. mw\_spontaneous\_Past\_Oriented\_TOT |  | Pearson's r |  | 0.279 | \*\*\* | -0.017 |  | 0.043 |  | 0.103 |  | 0.662 | \*\*\* | 0.023 |  | 0.437 | \*\*\* | 0.036 |  | -0.020 |  | 0.061 |  | 0.030 |  | -0.023 |  | 0.199 | \*\* | -0.005 |  | -0.007 |  | — |  |  |  |  |  |  |  |  |  |  |  |  |  |  |  |  |  |  |  |  |  |  |  |  |  |  |  |  |  |  |  |  |  |
|  |  | p-value |  | < .001 |  | 0.797 |  | 0.522 |  | 0.121 |  | < .001 |  | 0.731 |  | < .001 |  | 0.586 |  | 0.760 |  | 0.364 |  | 0.650 |  | 0.732 |  | 0.003 |  | 0.946 |  | 0.921 |  | — |  |  |  |  |  |  |  |  |  |  |  |  |  |  |  |  |  |  |  |  |  |  |  |  |  |  |  |  |  |  |  |  |  |
|  |  | Upper 95% CI |  | 0.395 |  | 0.114 |  | 0.172 |  | 0.231 |  | 0.729 |  | 0.153 |  | 0.537 |  | 0.166 |  | 0.110 |  | 0.190 |  | 0.160 |  | 0.108 |  | 0.321 |  | 0.126 |  | 0.124 |  | — |  |  |  |  |  |  |  |  |  |  |  |  |  |  |  |  |  |  |  |  |  |  |  |  |  |  |  |  |  |  |  |  |  |
|  |  | Lower 95% CI |  | 0.154 |  | -0.147 |  | -0.088 |  | -0.027 |  | 0.582 |  | -0.108 |  | 0.325 |  | -0.095 |  | -0.151 |  | -0.070 |  | -0.101 |  | -0.153 |  | 0.070 |  | -0.135 |  | -0.137 |  | — |  |  |  |  |  |  |  |  |  |  |  |  |  |  |  |  |  |  |  |  |  |  |  |  |  |  |  |  |  |  |  |  |  |
| 17. mw\_externe\_Past\_Oriented\_TOT |  | Pearson's r |  | 0.344 | \*\*\* | -0.023 |  | 0.112 |  | 0.067 |  | 0.593 | \*\*\* | 0.206 | \*\* | 0.119 |  | 0.471 | \*\*\* | 0.023 |  | 0.054 |  | 0.087 |  | 0.061 |  | 0.056 |  | 0.128 |  | -0.066 |  | 0.016 |  | — |  |  |  |  |  |  |  |  |  |  |  |  |  |  |  |  |  |  |  |  |  |  |  |  |  |  |  |  |  |  |  |
|  |  | p-value |  | < .001 |  | 0.735 |  | 0.093 |  | 0.316 |  | < .001 |  | 0.002 |  | 0.074 |  | < .001 |  | 0.733 |  | 0.416 |  | 0.195 |  | 0.358 |  | 0.401 |  | 0.054 |  | 0.325 |  | 0.813 |  | — |  |  |  |  |  |  |  |  |  |  |  |  |  |  |  |  |  |  |  |  |  |  |  |  |  |  |  |  |  |  |  |
|  |  | Upper 95% CI |  | 0.454 |  | 0.108 |  | 0.239 |  | 0.196 |  | 0.671 |  | 0.327 |  | 0.246 |  | 0.567 |  | 0.153 |  | 0.184 |  | 0.215 |  | 0.190 |  | 0.185 |  | 0.254 |  | 0.065 |  | 0.146 |  | — |  |  |  |  |  |  |  |  |  |  |  |  |  |  |  |  |  |  |  |  |  |  |  |  |  |  |  |  |  |  |  |
|  |  | Lower 95% CI |  | 0.223 |  | -0.153 |  | -0.019 |  | -0.064 |  | 0.501 |  | 0.077 |  | -0.011 |  | 0.363 |  | -0.108 |  | -0.077 |  | -0.044 |  | -0.070 |  | -0.075 |  | -0.002 |  | -0.195 |  | -0.115 |  | — |  |  |  |  |  |  |  |  |  |  |  |  |  |  |  |  |  |  |  |  |  |  |  |  |  |  |  |  |  |  |  |
| 18. mw\_voluntary\_Past\_Oriented\_TOT |  | Pearson's r |  | 0.124 |  | -0.075 |  | -9.910e -4 |  | -0.037 |  | 0.535 | \*\*\* | 0.057 |  | -0.073 |  | -0.011 |  | 0.408 | \*\*\* | -0.066 |  | -0.050 |  | 0.132 | \* | -0.108 |  | -0.022 |  | 0.059 |  | 0.034 |  | 0.074 |  | — |  |  |  |  |  |  |  |  |  |  |  |  |  |  |  |  |  |  |  |  |  |  |  |  |  |  |  |  |  |
|  |  | p-value |  | 0.064 |  | 0.261 |  | 0.988 |  | 0.579 |  | < .001 |  | 0.391 |  | 0.273 |  | 0.865 |  | < .001 |  | 0.320 |  | 0.453 |  | 0.048 |  | 0.106 |  | 0.740 |  | 0.381 |  | 0.616 |  | 0.265 |  | — |  |  |  |  |  |  |  |  |  |  |  |  |  |  |  |  |  |  |  |  |  |  |  |  |  |  |  |  |  |
|  |  | Upper 95% CI |  | 0.250 |  | 0.056 |  | 0.130 |  | 0.094 |  | 0.622 |  | 0.186 |  | 0.058 |  | 0.119 |  | 0.511 |  | 0.065 |  | 0.081 |  | 0.258 |  | 0.023 |  | 0.109 |  | 0.188 |  | 0.163 |  | 0.203 |  | — |  |  |  |  |  |  |  |  |  |  |  |  |  |  |  |  |  |  |  |  |  |  |  |  |  |  |  |  |  |
|  |  | Lower 95% CI |  | -0.007 |  | -0.204 |  | -0.131 |  | -0.167 |  | 0.435 |  | -0.074 |  | -0.202 |  | -0.142 |  | 0.293 |  | -0.195 |  | -0.179 |  | 0.001 |  | -0.235 |  | -0.152 |  | -0.073 |  | -0.097 |  | -0.057 |  | — |  |  |  |  |  |  |  |  |  |  |  |  |  |  |  |  |  |  |  |  |  |  |  |  |  |  |  |  |  |
| 19. mw\_spontaneous\_Imagination\_TOT |  | Pearson's r |  | 0.279 | \*\*\* | -0.017 |  | 0.043 |  | 0.103 |  | 0.662 | \*\*\* | 0.023 |  | 0.437 | \*\*\* | 0.036 |  | -0.020 |  | 0.061 |  | 0.030 |  | -0.023 |  | 0.199 | \*\* | -0.005 |  | -0.007 |  | 1.000 | \*\*\* | 0.016 |  | 0.034 |  | — |  |  |  |  |  |  |  |  |  |  |  |  |  |  |  |  |  |  |  |  |  |  |  |  |  |  |  |
|  |  | p-value |  | < .001 |  | 0.797 |  | 0.522 |  | 0.121 |  | < .001 |  | 0.731 |  | < .001 |  | 0.586 |  | 0.760 |  | 0.364 |  | 0.650 |  | 0.732 |  | 0.003 |  | 0.946 |  | 0.921 |  | < .001 |  | 0.813 |  | 0.616 |  | — |  |  |  |  |  |  |  |  |  |  |  |  |  |  |  |  |  |  |  |  |  |  |  |  |  |  |  |
|  |  | Upper 95% CI |  | 0.395 |  | 0.114 |  | 0.172 |  | 0.231 |  | 0.729 |  | 0.153 |  | 0.537 |  | 0.166 |  | 0.110 |  | 0.190 |  | 0.160 |  | 0.108 |  | 0.321 |  | 0.126 |  | 0.124 |  | 1.000 |  | 0.146 |  | 0.163 |  | — |  |  |  |  |  |  |  |  |  |  |  |  |  |  |  |  |  |  |  |  |  |  |  |  |  |  |  |
|  |  | Lower 95% CI |  | 0.154 |  | -0.147 |  | -0.088 |  | -0.027 |  | 0.582 |  | -0.108 |  | 0.325 |  | -0.095 |  | -0.151 |  | -0.070 |  | -0.101 |  | -0.153 |  | 0.070 |  | -0.135 |  | -0.137 |  | 1.000 |  | -0.115 |  | -0.097 |  | — |  |  |  |  |  |  |  |  |  |  |  |  |  |  |  |  |  |  |  |  |  |  |  |  |  |  |  |
| 20. mw\_externe\_Imagination\_TOT |  | Pearson's r |  | 0.344 | \*\*\* | -0.023 |  | 0.112 |  | 0.067 |  | 0.593 | \*\*\* | 0.206 | \*\* | 0.119 |  | 0.471 | \*\*\* | 0.023 |  | 0.054 |  | 0.087 |  | 0.061 |  | 0.056 |  | 0.128 |  | -0.066 |  | 0.016 |  | 1.000 | \*\*\* | 0.074 |  | 0.016 |  | — |  |  |  |  |  |  |  |  |  |  |  |  |  |  |  |  |  |  |  |  |  |  |  |  |  |
|  |  | p-value |  | < .001 |  | 0.735 |  | 0.093 |  | 0.316 |  | < .001 |  | 0.002 |  | 0.074 |  | < .001 |  | 0.733 |  | 0.416 |  | 0.195 |  | 0.358 |  | 0.401 |  | 0.054 |  | 0.325 |  | 0.813 |  | < .001 |  | 0.265 |  | 0.813 |  | — |  |  |  |  |  |  |  |  |  |  |  |  |  |  |  |  |  |  |  |  |  |  |  |  |  |
|  |  | Upper 95% CI |  | 0.454 |  | 0.108 |  | 0.239 |  | 0.196 |  | 0.671 |  | 0.327 |  | 0.246 |  | 0.567 |  | 0.153 |  | 0.184 |  | 0.215 |  | 0.190 |  | 0.185 |  | 0.254 |  | 0.065 |  | 0.146 |  | 1.000 |  | 0.203 |  | 0.146 |  | — |  |  |  |  |  |  |  |  |  |  |  |  |  |  |  |  |  |  |  |  |  |  |  |  |  |
|  |  | Lower 95% CI |  | 0.223 |  | -0.153 |  | -0.019 |  | -0.064 |  | 0.501 |  | 0.077 |  | -0.011 |  | 0.363 |  | -0.108 |  | -0.077 |  | -0.044 |  | -0.070 |  | -0.075 |  | -0.002 |  | -0.195 |  | -0.115 |  | 1.000 |  | -0.057 |  | -0.115 |  | — |  |  |  |  |  |  |  |  |  |  |  |  |  |  |  |  |  |  |  |  |  |  |  |  |  |
| 21. mw\_voluntary\_Imagination\_TOT |  | Pearson's r |  | 0.124 |  | -0.075 |  | -9.910e -4 |  | -0.037 |  | 0.535 | \*\*\* | 0.057 |  | -0.073 |  | -0.011 |  | 0.408 | \*\*\* | -0.066 |  | -0.050 |  | 0.132 | \* | -0.108 |  | -0.022 |  | 0.059 |  | 0.034 |  | 0.074 |  | 1.000 | \*\*\* | 0.034 |  | 0.074 |  | — |  |  |  |  |  |  |  |  |  |  |  |  |  |  |  |  |  |  |  |  |  |  |  |
|  |  | p-value |  | 0.064 |  | 0.261 |  | 0.988 |  | 0.579 |  | < .001 |  | 0.391 |  | 0.273 |  | 0.865 |  | < .001 |  | 0.320 |  | 0.453 |  | 0.048 |  | 0.106 |  | 0.740 |  | 0.381 |  | 0.616 |  | 0.265 |  | < .001 |  | 0.616 |  | 0.265 |  | — |  |  |  |  |  |  |  |  |  |  |  |  |  |  |  |  |  |  |  |  |  |  |  |
|  |  | Upper 95% CI |  | 0.250 |  | 0.056 |  | 0.130 |  | 0.094 |  | 0.622 |  | 0.186 |  | 0.058 |  | 0.119 |  | 0.511 |  | 0.065 |  | 0.081 |  | 0.258 |  | 0.023 |  | 0.109 |  | 0.188 |  | 0.163 |  | 0.203 |  | 1.000 |  | 0.163 |  | 0.203 |  | — |  |  |  |  |  |  |  |  |  |  |  |  |  |  |  |  |  |  |  |  |  |  |  |
|  |  | Lower 95% CI |  | -0.007 |  | -0.204 |  | -0.131 |  | -0.167 |  | 0.435 |  | -0.074 |  | -0.202 |  | -0.142 |  | 0.293 |  | -0.195 |  | -0.179 |  | 0.001 |  | -0.235 |  | -0.152 |  | -0.073 |  | -0.097 |  | -0.057 |  | 1.000 |  | -0.097 |  | -0.057 |  | — |  |  |  |  |  |  |  |  |  |  |  |  |  |  |  |  |  |  |  |  |  |  |  |
| 22. Total\_MWQ |  | Pearson's r |  | 0.232 | \*\* | 0.052 |  | 0.228 | \*\* | 0.049 |  | -0.020 |  | 0.169 | \* | 0.256 | \*\* | 0.163 | \* | -0.098 |  | 0.269 | \*\*\* | 0.133 |  | -0.029 |  | 0.172 | \* | 0.053 |  | -0.122 |  | -0.133 |  | 0.122 |  | -0.010 |  | -0.133 |  | 0.122 |  | -0.010 |  | — |  |  |  |  |  |  |  |  |  |  |  |  |  |  |  |  |  |  |  |  |  |
|  |  | p-value |  | 0.004 |  | 0.529 |  | 0.005 |  | 0.551 |  | 0.807 |  | 0.037 |  | 0.001 |  | 0.045 |  | 0.228 |  | < .001 |  | 0.101 |  | 0.724 |  | 0.034 |  | 0.520 |  | 0.134 |  | 0.104 |  | 0.134 |  | 0.900 |  | 0.104 |  | 0.134 |  | 0.900 |  | — |  |  |  |  |  |  |  |  |  |  |  |  |  |  |  |  |  |  |  |  |  |
|  |  | Upper 95% CI |  | 0.377 |  | 0.209 |  | 0.373 |  | 0.206 |  | 0.140 |  | 0.320 |  | 0.399 |  | 0.314 |  | 0.062 |  | 0.411 |  | 0.287 |  | 0.131 |  | 0.322 |  | 0.210 |  | 0.038 |  | 0.027 |  | 0.276 |  | 0.149 |  | 0.027 |  | 0.276 |  | 0.149 |  | — |  |  |  |  |  |  |  |  |  |  |  |  |  |  |  |  |  |  |  |  |  |
|  |  | Lower 95% CI |  | 0.076 |  | -0.109 |  | 0.071 |  | -0.111 |  | -0.179 |  | 0.010 |  | 0.101 |  | 0.004 |  | -0.254 |  | 0.115 |  | -0.026 |  | -0.187 |  | 0.013 |  | -0.108 |  | -0.276 |  | -0.286 |  | -0.038 |  | -0.169 |  | -0.286 |  | -0.038 |  | -0.169 |  | — |  |  |  |  |  |  |  |  |  |  |  |  |  |  |  |  |  |  |  |  |  |
| 23. Correct\_ratio |  | Pearson's r |  | 0.039 |  | -0.008 |  | -0.004 |  | 0.009 |  | 0.102 |  | 0.020 |  | 0.051 |  | 0.026 |  | -0.009 |  | -0.032 |  | 0.024 |  | 0.005 |  | -0.006 |  | 0.023 |  | -0.002 |  | 0.123 |  | 0.040 |  | 0.003 |  | 0.123 |  | 0.040 |  | 0.003 |  | -0.186 | \* | — |  |  |  |  |  |  |  |  |  |  |  |  |  |  |  |  |  |  |  |
|  |  | p-value |  | 0.560 |  | 0.909 |  | 0.947 |  | 0.889 |  | 0.126 |  | 0.765 |  | 0.449 |  | 0.693 |  | 0.890 |  | 0.637 |  | 0.720 |  | 0.939 |  | 0.929 |  | 0.731 |  | 0.979 |  | 0.064 |  | 0.552 |  | 0.959 |  | 0.064 |  | 0.552 |  | 0.959 |  | 0.022 |  | — |  |  |  |  |  |  |  |  |  |  |  |  |  |  |  |  |  |  |  |
|  |  | Upper 95% CI |  | 0.169 |  | 0.123 |  | 0.126 |  | 0.140 |  | 0.230 |  | 0.150 |  | 0.180 |  | 0.156 |  | 0.121 |  | 0.099 |  | 0.154 |  | 0.135 |  | 0.125 |  | 0.153 |  | 0.129 |  | 0.250 |  | 0.169 |  | 0.134 |  | 0.250 |  | 0.169 |  | 0.134 |  | -0.028 |  | — |  |  |  |  |  |  |  |  |  |  |  |  |  |  |  |  |  |  |  |
|  |  | Lower 95% CI |  | -0.092 |  | -0.138 |  | -0.135 |  | -0.121 |  | -0.029 |  | -0.111 |  | -0.080 |  | -0.104 |  | -0.140 |  | -0.161 |  | -0.107 |  | -0.126 |  | -0.136 |  | -0.108 |  | -0.132 |  | -0.007 |  | -0.091 |  | -0.127 |  | -0.007 |  | -0.091 |  | -0.127 |  | -0.335 |  | — |  |  |  |  |  |  |  |  |  |  |  |  |  |  |  |  |  |  |  |
| 24. Incorrect\_ratio |  | Pearson's r |  | -0.020 |  | -0.018 |  | 0.009 |  | -0.008 |  | 0.040 |  | -0.051 |  | 0.107 |  | -0.064 |  | -0.085 |  | 0.062 |  | -0.053 |  | -0.003 |  | 0.051 |  | 0.031 |  | -0.089 |  | 0.136 | \* | -0.063 |  | -0.024 |  | 0.136 | \* | -0.063 |  | -0.024 |  | -0.188 | \* | -0.037 |  | — |  |  |  |  |  |  |  |  |  |  |  |  |  |  |  |  |  |
|  |  | p-value |  | 0.770 |  | 0.782 |  | 0.887 |  | 0.903 |  | 0.546 |  | 0.445 |  | 0.109 |  | 0.336 |  | 0.204 |  | 0.354 |  | 0.431 |  | 0.965 |  | 0.448 |  | 0.642 |  | 0.181 |  | 0.041 |  | 0.343 |  | 0.715 |  | 0.041 |  | 0.343 |  | 0.715 |  | 0.020 |  | 0.576 |  | — |  |  |  |  |  |  |  |  |  |  |  |  |  |  |  |  |  |
|  |  | Upper 95% CI |  | 0.111 |  | 0.112 |  | 0.140 |  | 0.122 |  | 0.170 |  | 0.080 |  | 0.234 |  | 0.067 |  | 0.046 |  | 0.191 |  | 0.078 |  | 0.128 |  | 0.180 |  | 0.161 |  | 0.042 |  | 0.262 |  | 0.068 |  | 0.106 |  | 0.262 |  | 0.068 |  | 0.106 |  | -0.030 |  | 0.094 |  | — |  |  |  |  |  |  |  |  |  |  |  |  |  |  |  |  |  |
|  |  | Lower 95% CI |  | -0.150 |  | -0.149 |  | -0.121 |  | -0.139 |  | -0.091 |  | -0.180 |  | -0.024 |  | -0.193 |  | -0.213 |  | -0.069 |  | -0.182 |  | -0.133 |  | -0.080 |  | -0.100 |  | -0.217 |  | 0.006 |  | -0.192 |  | -0.154 |  | 0.006 |  | -0.192 |  | -0.154 |  | -0.337 |  | -0.167 |  | — |  |  |  |  |  |  |  |  |  |  |  |  |  |  |  |  |  |
| 25. Omission\_ratio |  | Pearson's r |  | -0.016 |  | 0.019 |  | -0.003 |  | -0.001 |  | -0.104 |  | 0.021 |  | -0.112 |  | 0.025 |  | 0.066 |  | -0.019 |  | 0.019 |  | -0.002 |  | -0.031 |  | -0.039 |  | 0.063 |  | -0.186 | \*\* | 0.014 |  | 0.014 |  | -0.186 | \*\* | 0.014 |  | 0.014 |  | 0.272 | \*\*\* | -0.721 | \*\*\* | -0.666 | \*\*\* | — |  |  |  |  |  |  |  |  |  |  |  |  |  |  |  |
|  |  | p-value |  | 0.816 |  | 0.782 |  | 0.961 |  | 0.985 |  | 0.118 |  | 0.759 |  | 0.093 |  | 0.710 |  | 0.325 |  | 0.771 |  | 0.781 |  | 0.979 |  | 0.645 |  | 0.562 |  | 0.344 |  | 0.005 |  | 0.831 |  | 0.830 |  | 0.005 |  | 0.831 |  | 0.830 |  | < .001 |  | < .001 |  | < .001 |  | — |  |  |  |  |  |  |  |  |  |  |  |  |  |  |  |
|  |  | Upper 95% CI |  | 0.115 |  | 0.149 |  | 0.127 |  | 0.129 |  | 0.027 |  | 0.151 |  | 0.019 |  | 0.155 |  | 0.195 |  | 0.111 |  | 0.149 |  | 0.129 |  | 0.100 |  | 0.092 |  | 0.192 |  | -0.057 |  | 0.145 |  | 0.145 |  | -0.057 |  | 0.145 |  | 0.145 |  | 0.413 |  | -0.651 |  | -0.586 |  | — |  |  |  |  |  |  |  |  |  |  |  |  |  |  |  |
|  |  | Lower 95% CI |  | -0.146 |  | -0.112 |  | -0.134 |  | -0.132 |  | -0.232 |  | -0.110 |  | -0.239 |  | -0.106 |  | -0.065 |  | -0.150 |  | -0.112 |  | -0.132 |  | -0.161 |  | -0.168 |  | -0.068 |  | -0.309 |  | -0.116 |  | -0.116 |  | -0.309 |  | -0.116 |  | -0.116 |  | 0.118 |  | -0.778 |  | -0.733 |  | — |  |  |  |  |  |  |  |  |  |  |  |  |  |  |  |
| 26. Meta\_Aptitude |  | Pearson's r |  | -0.164 | \* | -0.084 |  | -0.103 |  | -0.044 |  | -0.094 |  | -0.064 |  | -0.157 | \* | -0.016 |  | -0.040 |  | -0.119 |  | -0.019 |  | -0.033 |  | -0.144 | \* | 0.030 |  | 0.026 |  | 0.041 |  | -0.121 |  | -0.116 |  | 0.041 |  | -0.121 |  | -0.116 |  | -0.299 | \*\*\* | 0.209 | \*\* | 0.006 |  | -0.164 | \* | — |  |  |  |  |  |  |  |  |  |  |  |  |  |
|  |  | p-value |  | 0.019 |  | 0.234 |  | 0.143 |  | 0.528 |  | 0.181 |  | 0.365 |  | 0.025 |  | 0.817 |  | 0.567 |  | 0.089 |  | 0.792 |  | 0.642 |  | 0.040 |  | 0.666 |  | 0.712 |  | 0.560 |  | 0.083 |  | 0.100 |  | 0.560 |  | 0.083 |  | 0.100 |  | < .001 |  | 0.003 |  | 0.934 |  | 0.019 |  | — |  |  |  |  |  |  |  |  |  |  |  |  |  |
|  |  | Upper 95% CI |  | -0.028 |  | 0.054 |  | 0.035 |  | 0.094 |  | 0.044 |  | 0.074 |  | -0.020 |  | 0.121 |  | 0.098 |  | 0.018 |  | 0.119 |  | 0.105 |  | -0.007 |  | 0.167 |  | 0.163 |  | 0.177 |  | 0.016 |  | 0.022 |  | 0.177 |  | 0.016 |  | 0.022 |  | -0.138 |  | 0.337 |  | 0.143 |  | -0.027 |  | — |  |  |  |  |  |  |  |  |  |  |  |  |  |
|  |  | Lower 95% CI |  | -0.295 |  | -0.218 |  | -0.237 |  | -0.181 |  | -0.228 |  | -0.199 |  | -0.288 |  | -0.153 |  | -0.177 |  | -0.253 |  | -0.156 |  | -0.169 |  | -0.276 |  | -0.107 |  | -0.112 |  | -0.097 |  | -0.255 |  | -0.249 |  | -0.097 |  | -0.255 |  | -0.249 |  | -0.444 |  | 0.074 |  | -0.132 |  | -0.294 |  | — |  |  |  |  |  |  |  |  |  |  |  |  |  |
| 27. Meta\_Internal |  | Pearson's r |  | 0.062 |  | -0.152 | \* | 0.135 |  | 0.072 |  | -2.236e -4 |  | 0.043 |  | 0.082 |  | 0.057 |  | 0.079 |  | 0.041 |  | 0.113 |  | 0.089 |  | 0.031 |  | 0.040 |  | 0.052 |  | 0.099 |  | -0.095 |  | -0.026 |  | 0.099 |  | -0.095 |  | -0.026 |  | -0.034 |  | 0.043 |  | 0.030 |  | -0.054 |  | 0.252 | \*\*\* | — |  |  |  |  |  |  |  |  |  |  |  |
|  |  | p-value |  | 0.380 |  | 0.030 |  | 0.055 |  | 0.308 |  | 0.997 |  | 0.538 |  | 0.245 |  | 0.421 |  | 0.260 |  | 0.563 |  | 0.109 |  | 0.206 |  | 0.656 |  | 0.573 |  | 0.457 |  | 0.159 |  | 0.175 |  | 0.713 |  | 0.159 |  | 0.175 |  | 0.713 |  | 0.697 |  | 0.545 |  | 0.669 |  | 0.446 |  | < .001 |  | — |  |  |  |  |  |  |  |  |  |  |  |
|  |  | Upper 95% CI |  | 0.197 |  | -0.015 |  | 0.267 |  | 0.207 |  | 0.137 |  | 0.180 |  | 0.217 |  | 0.193 |  | 0.214 |  | 0.177 |  | 0.246 |  | 0.224 |  | 0.168 |  | 0.176 |  | 0.188 |  | 0.233 |  | 0.043 |  | 0.112 |  | 0.233 |  | 0.043 |  | 0.112 |  | 0.135 |  | 0.179 |  | 0.167 |  | 0.084 |  | 0.376 |  | — |  |  |  |  |  |  |  |  |  |  |  |
|  |  | Lower 95% CI |  | -0.076 |  | -0.284 |  | -0.003 |  | -0.066 |  | -0.138 |  | -0.095 |  | -0.056 |  | -0.081 |  | -0.059 |  | -0.097 |  | -0.025 |  | -0.049 |  | -0.106 |  | -0.098 |  | -0.086 |  | -0.039 |  | -0.230 |  | -0.163 |  | -0.039 |  | -0.230 |  | -0.163 |  | -0.200 |  | -0.095 |  | -0.108 |  | -0.190 |  | 0.119 |  | — |  |  |  |  |  |  |  |  |  |  |  |
| 28. Meta\_External |  | Pearson's r |  | 0.032 |  | -0.063 |  | 0.003 |  | 0.018 |  | 0.022 |  | 0.087 |  | 0.049 |  | 0.124 |  | -0.085 |  | -0.082 |  | 0.116 |  | -0.019 |  | 0.056 |  | 0.047 |  | -0.066 |  | 0.062 |  | 0.066 |  | -0.111 |  | 0.062 |  | 0.066 |  | -0.111 |  | 0.125 |  | -0.008 |  | 0.006 |  | 0.002 |  | -0.094 |  | 0.205 | \*\* | — |  |  |  |  |  |  |  |  |  |
|  |  | p-value |  | 0.648 |  | 0.371 |  | 0.964 |  | 0.797 |  | 0.755 |  | 0.216 |  | 0.483 |  | 0.077 |  | 0.229 |  | 0.246 |  | 0.099 |  | 0.792 |  | 0.429 |  | 0.501 |  | 0.349 |  | 0.375 |  | 0.346 |  | 0.113 |  | 0.375 |  | 0.346 |  | 0.113 |  | 0.147 |  | 0.909 |  | 0.934 |  | 0.977 |  | 0.180 |  | 0.003 |  | — |  |  |  |  |  |  |  |  |  |
|  |  | Upper 95% CI |  | 0.169 |  | 0.075 |  | 0.140 |  | 0.155 |  | 0.159 |  | 0.222 |  | 0.186 |  | 0.257 |  | 0.053 |  | 0.056 |  | 0.249 |  | 0.119 |  | 0.192 |  | 0.184 |  | 0.072 |  | 0.198 |  | 0.202 |  | 0.026 |  | 0.198 |  | 0.202 |  | 0.026 |  | 0.286 |  | 0.129 |  | 0.143 |  | 0.139 |  | 0.044 |  | 0.333 |  | — |  |  |  |  |  |  |  |  |  |
|  |  | Lower 95% CI |  | -0.106 |  | -0.199 |  | -0.134 |  | -0.120 |  | -0.116 |  | -0.051 |  | -0.089 |  | -0.014 |  | -0.219 |  | -0.216 |  | -0.022 |  | -0.156 |  | -0.082 |  | -0.091 |  | -0.201 |  | -0.076 |  | -0.072 |  | -0.245 |  | -0.076 |  | -0.072 |  | -0.245 |  | -0.044 |  | -0.145 |  | -0.132 |  | -0.135 |  | -0.229 |  | 0.070 |  | — |  |  |  |  |  |  |  |  |  |
| 29. Age |  | Pearson's r |  | -0.161 | \* | -0.055 |  | -0.224 | \*\*\* | 0.084 |  | 0.010 |  | -0.223 | \*\*\* | -0.145 | \* | -0.074 |  | -0.011 |  | -0.116 |  | -0.232 | \*\*\* | -0.052 |  | -0.047 |  | 0.124 |  | 0.058 |  | 0.052 |  | -0.008 |  | -0.040 |  | 0.052 |  | -0.008 |  | -0.040 |  | -0.322 | \*\*\* | 0.066 |  | 0.089 |  | -0.111 |  | 0.201 | \*\* | 0.030 |  | -0.093 |  | — |  |  |  |  |  |  |  |
|  |  | p-value |  | 0.015 |  | 0.410 |  | < .001 |  | 0.210 |  | 0.880 |  | < .001 |  | 0.030 |  | 0.268 |  | 0.869 |  | 0.082 |  | < .001 |  | 0.441 |  | 0.486 |  | 0.063 |  | 0.385 |  | 0.440 |  | 0.911 |  | 0.554 |  | 0.440 |  | 0.911 |  | 0.554 |  | < .001 |  | 0.324 |  | 0.183 |  | 0.096 |  | 0.004 |  | 0.674 |  | 0.186 |  | — |  |  |  |  |  |  |  |
|  |  | Upper 95% CI |  | -0.031 |  | 0.076 |  | -0.096 |  | 0.212 |  | 0.140 |  | -0.095 |  | -0.015 |  | 0.057 |  | 0.120 |  | 0.015 |  | -0.105 |  | 0.079 |  | 0.084 |  | 0.250 |  | 0.187 |  | 0.181 |  | 0.123 |  | 0.091 |  | 0.181 |  | 0.123 |  | 0.091 |  | -0.172 |  | 0.195 |  | 0.217 |  | 0.020 |  | 0.329 |  | 0.166 |  | 0.045 |  | — |  |  |  |  |  |  |  |
|  |  | Lower 95% CI |  | -0.286 |  | -0.184 |  | -0.344 |  | -0.047 |  | -0.121 |  | -0.343 |  | -0.270 |  | -0.203 |  | -0.141 |  | -0.243 |  | -0.352 |  | -0.181 |  | -0.176 |  | -0.007 |  | -0.073 |  | -0.079 |  | -0.138 |  | -0.169 |  | -0.079 |  | -0.138 |  | -0.169 |  | -0.458 |  | -0.065 |  | -0.042 |  | -0.238 |  | 0.065 |  | -0.108 |  | -0.227 |  | — |  |  |  |  |  |  |  |
| 30. Expert |  | Pearson's r |  | -0.171 | \* | -0.026 |  | -0.192 | \*\* | 0.006 |  | -0.005 |  | -0.178 | \*\* | -0.147 | \* | -0.022 |  | -0.108 |  | -0.107 |  | -0.127 |  | -0.111 |  | -0.069 |  | 0.105 |  | -0.031 |  | 0.046 |  | -0.046 |  | -0.019 |  | 0.046 |  | -0.046 |  | -0.019 |  | -0.259 | \*\* | 0.033 |  | 0.050 |  | -0.059 |  | 0.180 | \*\* | -0.026 |  | 0.022 |  | 0.450 | \*\*\* | — |  |  |  |  |  |
|  |  | p-value |  | 0.010 |  | 0.697 |  | 0.004 |  | 0.934 |  | 0.939 |  | 0.007 |  | 0.027 |  | 0.743 |  | 0.105 |  | 0.109 |  | 0.057 |  | 0.096 |  | 0.302 |  | 0.114 |  | 0.646 |  | 0.494 |  | 0.491 |  | 0.775 |  | 0.494 |  | 0.491 |  | 0.775 |  | 0.001 |  | 0.618 |  | 0.458 |  | 0.374 |  | 0.010 |  | 0.717 |  | 0.758 |  | < .001 |  | — |  |  |  |  |  |
|  |  | Upper 95% CI |  | -0.041 |  | 0.105 |  | -0.063 |  | 0.136 |  | 0.125 |  | -0.049 |  | -0.017 |  | 0.109 |  | 0.023 |  | 0.024 |  | 0.004 |  | 0.020 |  | 0.062 |  | 0.233 |  | 0.100 |  | 0.175 |  | 0.085 |  | 0.112 |  | 0.175 |  | 0.085 |  | 0.112 |  | -0.104 |  | 0.163 |  | 0.179 |  | 0.072 |  | 0.310 |  | 0.112 |  | 0.159 |  | 0.548 |  | — |  |  |  |  |  |
|  |  | Lower 95% CI |  | -0.295 |  | -0.156 |  | -0.315 |  | -0.125 |  | -0.136 |  | -0.302 |  | -0.273 |  | -0.152 |  | -0.235 |  | -0.234 |  | -0.253 |  | -0.238 |  | -0.198 |  | -0.025 |  | -0.161 |  | -0.085 |  | -0.176 |  | -0.149 |  | -0.085 |  | -0.176 |  | -0.149 |  | -0.402 |  | -0.098 |  | -0.081 |  | -0.188 |  | 0.044 |  | -0.162 |  | -0.116 |  | 0.340 |  | — |  |  |  |  |  |
| 31. PHQ4\_Depression |  | Pearson's r |  | 0.058 |  | -0.116 |  | 0.124 |  | 0.056 |  | -0.034 |  | 0.065 |  | 0.139 |  | -0.007 |  | 0.055 |  | 0.077 |  | -8.186e -4 |  | 0.147 |  | 0.134 |  | -0.038 |  | 0.012 |  | -0.052 |  | 0.023 |  | -0.029 |  | -0.052 |  | 0.023 |  | -0.029 |  | 0.239 | \*\* | -0.063 |  | 0.014 |  | 0.036 |  | -0.073 |  | -0.082 |  | 0.160 |  | -0.081 |  | -0.081 |  | — |  |  |  |
|  |  | p-value |  | 0.475 |  | 0.154 |  | 0.129 |  | 0.490 |  | 0.679 |  | 0.426 |  | 0.087 |  | 0.936 |  | 0.499 |  | 0.347 |  | 0.992 |  | 0.071 |  | 0.099 |  | 0.646 |  | 0.880 |  | 0.523 |  | 0.781 |  | 0.720 |  | 0.523 |  | 0.781 |  | 0.720 |  | 0.003 |  | 0.438 |  | 0.867 |  | 0.658 |  | 0.395 |  | 0.341 |  | 0.061 |  | 0.320 |  | 0.319 |  | — |  |  |  |
|  |  | Upper 95% CI |  | 0.216 |  | 0.044 |  | 0.277 |  | 0.214 |  | 0.126 |  | 0.222 |  | 0.292 |  | 0.153 |  | 0.213 |  | 0.233 |  | 0.158 |  | 0.299 |  | 0.287 |  | 0.122 |  | 0.171 |  | 0.108 |  | 0.181 |  | 0.131 |  | 0.108 |  | 0.181 |  | 0.131 |  | 0.384 |  | 0.097 |  | 0.173 |  | 0.194 |  | 0.096 |  | 0.087 |  | 0.319 |  | 0.079 |  | 0.079 |  | — |  |  |  |
|  |  | Lower 95% CI |  | -0.102 |  | -0.270 |  | -0.036 |  | -0.104 |  | -0.192 |  | -0.095 |  | -0.020 |  | -0.166 |  | -0.105 |  | -0.083 |  | -0.160 |  | -0.013 |  | -0.026 |  | -0.196 |  | -0.147 |  | -0.210 |  | -0.137 |  | -0.188 |  | -0.210 |  | -0.137 |  | -0.188 |  | 0.083 |  | -0.220 |  | -0.146 |  | -0.124 |  | -0.238 |  | -0.246 |  | -0.008 |  | -0.237 |  | -0.237 |  | — |  |  |  |
| 32. PHQ4\_Anxiety |  | Pearson's r |  | 0.074 |  | -0.214 | \*\* | 0.090 |  | 0.140 |  | -0.068 |  | 0.131 |  | 0.105 |  | 0.091 |  | 0.099 |  | 0.057 |  | -0.015 |  | 0.122 |  | 0.094 |  | 0.090 |  | 0.054 |  | -0.055 |  | -0.047 |  | -0.016 |  | -0.055 |  | -0.047 |  | -0.016 |  | 0.158 |  | -0.063 |  | -0.049 |  | 0.081 |  | -0.053 |  | 0.154 |  | 0.110 |  | -0.087 |  | -0.153 |  | 0.649 | \*\*\* | — |  |
|  |  | p-value |  | 0.363 |  | 0.008 |  | 0.269 |  | 0.085 |  | 0.402 |  | 0.108 |  | 0.197 |  | 0.266 |  | 0.226 |  | 0.483 |  | 0.856 |  | 0.135 |  | 0.252 |  | 0.270 |  | 0.510 |  | 0.497 |  | 0.569 |  | 0.850 |  | 0.497 |  | 0.569 |  | 0.850 |  | 0.052 |  | 0.443 |  | 0.549 |  | 0.319 |  | 0.538 |  | 0.073 |  | 0.199 |  | 0.288 |  | 0.060 |  | < .001 |  | — |  |
|  |  | Upper 95% CI |  | 0.231 |  | -0.057 |  | 0.246 |  | 0.293 |  | 0.092 |  | 0.284 |  | 0.260 |  | 0.246 |  | 0.254 |  | 0.215 |  | 0.145 |  | 0.276 |  | 0.249 |  | 0.246 |  | 0.211 |  | 0.105 |  | 0.113 |  | 0.144 |  | 0.105 |  | 0.113 |  | 0.144 |  | 0.309 |  | 0.097 |  | 0.111 |  | 0.237 |  | 0.116 |  | 0.313 |  | 0.273 |  | 0.073 |  | 0.007 |  | 0.733 |  | — |  |
|  |  | Lower 95% CI |  | -0.086 |  | -0.361 |  | -0.070 |  | -0.019 |  | -0.225 |  | -0.029 |  | -0.055 |  | -0.069 |  | -0.061 |  | -0.103 |  | -0.174 |  | -0.038 |  | -0.067 |  | -0.070 |  | -0.106 |  | -0.213 |  | -0.204 |  | -0.174 |  | -0.213 |  | -0.204 |  | -0.174 |  | -0.002 |  | -0.220 |  | -0.207 |  | -0.079 |  | -0.219 |  | -0.014 |  | -0.058 |  | -0.243 |  | -0.304 |  | 0.547 |  | — |  |
|  | | | | | | | | | | | | | | | | | | | | | | | | | | | | | | | | | | | | | | | | | | | | | | | | | | | | | | | | | | | | | | | | | | | |
|  |  |  |  |  |  |  |  |  |  |  |  |  |  |  |  |  |  |  |  |  |  |  |  |  |  |  |  |  |  |  |  |  |  |  |  |  |  |  |  |  |  |  |  |  |  |  |  |  |  |  |  |  |  |  |  |  |  |  |  |  |  |  |  |  |  |  |  |
| --- | --- | --- | --- | --- | --- | --- | --- | --- | --- | --- | --- | --- | --- | --- | --- | --- | --- | --- | --- | --- | --- | --- | --- | --- | --- | --- | --- | --- | --- | --- | --- | --- | --- | --- | --- | --- | --- | --- | --- | --- | --- | --- | --- | --- | --- | --- | --- | --- | --- | --- | --- | --- | --- | --- | --- | --- | --- | --- | --- | --- | --- | --- | --- | --- | --- | --- | --- |
| \* p < .05, \*\* p < .01, \*\*\* p < .001 | | | | | | | | | | | | | | | | | | | | | | | | | | | | | | | | | | | | | | | | | | | | | | | | | | | | | | | | | | | | | | | | | | | |
