# Supplementary material for: Where Is My Mind…? The Link between Mind Wandering and Prospective Memory
Source: Brain Sci. 2022 Aug 26;12(9):1139. doi: 10.3390/brainsci12091139 (PMC9497275; doi:10.3390/brainsci12091139)
Supplement: Supplementary file 1 [file brainsci-12-01139-s001.zip › Supplementary_Materials/S2-Interaction-Post_Hoc.pdf]

Post Hoc Comparisons - Source MW ✻ Orientation

		Mean Difference	95% CI for Mean Difference Lower	Upper	SE	t	p_{bonf}
Spontaneous, EFT	External, EFT	0.009	−0.005	0.022	0.004	2.194	1.000
	Voluntary, EFT	0.013	−5.671e −4	0.026	0.004	3.232	0.083
	Spontaneous, Planning	−0.022	−0.034	−0.009	0.004	−5.829	< .001
	External, Planning	−0.018	−0.032	−0.005	0.004	−4.517	< .001
	Voluntary, Planning	−0.035	−0.048	−0.021	0.004	−8.587	< .001
	Spontaneous, Past	0.016	0.003	0.028	0.004	4.246	0.001
	External, Past	0.020	0.007	0.034	0.004	5.026	< .001
	Voluntary, Past	0.025	0.011	0.038	0.004	6.109	< .001
	Spontaneous, Imagination	0.016	0.003	0.028	0.004	4.246	0.001
	External, Imagination	0.020	0.007	0.034	0.004	5.026	< .001
	Voluntary, Imagination	0.025	0.011	0.038	0.004	6.109	< .001
External, EFT	Voluntary, EFT	0.004	−0.009	0.018	0.004	1.038	1.000
	Spontaneous, Planning	−0.030	−0.044	−0.017	0.004	−7.525	< .001
	External, Planning	−0.027	−0.039	−0.014	0.004	−7.291	< .001
	Voluntary, Planning	−0.043	−0.057	−0.030	0.004	−10.762	< .001
	Spontaneous, Past	0.007	−0.007	0.021	0.004	1.723	1.000
	External, Past	0.011	−9.877e −4	0.024	0.004	3.106	0.127
	Voluntary, Past	0.016	0.002	0.029	0.004	3.934	0.006
	Spontaneous, Imagination	0.007	−0.007	0.021	0.004	1.723	1.000
	External, Imagination	0.011	−9.877e −4	0.024	0.004	3.106	0.127
	Voluntary, Imagination	0.016	0.002	0.029	0.004	3.934	0.006
Voluntary, EFT	Spontaneous, Planning	−0.034	−0.048	−0.021	0.004	−8.554	< .001
	External, Planning	−0.031	−0.045	−0.018	0.004	−7.721	< .001
	Voluntary, Planning	−0.048	−0.060	−0.035	0.004	−12.845	< .001
	Spontaneous, Past	0.003	−0.011	0.016	0.004	0.694	1.000
	External, Past	0.007	−0.006	0.021	0.004	1.822	1.000
	Voluntary, Past	0.012	−7.683e −4	0.024	0.004	3.165	0.104
	Spontaneous, Imagination	0.003	−0.011	0.016	0.004	0.694	1.000
	External, Imagination	0.007	−0.006	0.021	0.004	1.822	1.000
	Voluntary, Imagination	0.012	−7.683e −4	0.024	0.004	3.165	0.104
Spontaneous, Planning	External, Planning	0.003	−0.010	0.017	0.004	0.840	1.000
	Voluntary, Planning	−0.013	−0.027	4.316e −4	0.004	−3.266	0.073
	Spontaneous, Past	0.037	0.025	0.050	0.004	10.075	< .001
	External, Past	0.042	0.028	0.055	0.004	10.376	< .001
	Voluntary, Past	0.046	0.033	0.060	0.004	11.459	< .001
	Spontaneous, Imagination	0.037	0.025	0.050	0.004	10.075	< .001
	External, Imagination	0.042	0.028	0.055	0.004	10.376	< .001
	Voluntary, Imagination	0.046	0.033	0.060	0.004	11.459	< .001
External, Planning	Voluntary, Planning	−0.016	−0.030	−0.003	0.004	−4.106	0.003
	Spontaneous, Past	0.034	0.020	0.048	0.004	8.415	< .001
	External, Past	0.038	0.026	0.051	0.004	10.397	< .001
	Voluntary, Past	0.043	0.029	0.056	0.004	10.626	< .001
	Spontaneous, Imagination	0.034	0.020	0.048	0.004	8.415	< .001
	External, Imagination	0.038	0.026	0.051	0.004	10.397	< .001
	Voluntary, Imagination	0.043	0.029	0.056	0.004	10.626	< .001
Voluntary, Planning	Spontaneous, Past	0.050	0.037	0.064	0.004	12.485	< .001
	External, Past	0.055	0.041	0.068	0.004	13.613	< .001
	Voluntary, Past	0.059	0.047	0.072	0.004	16.011	< .001
	Spontaneous, Imagination	0.050	0.037	0.064	0.004	12.485	< .001
	External, Imagination	0.055	0.041	0.068	0.004	13.613	< .001
	Voluntary, Imagination	0.059	0.047	0.072	0.004	16.011	< .001
Spontaneous, Past	External, Past	0.005	−0.009	0.018	0.004	1.138	1.000
	Voluntary, Past	0.009	−0.005	0.022	0.004	2.231	1.000
	Spontaneous, Imagination	6.282e −16	−0.012	0.012	0.004	1.697e −13	1.000
	External, Imagination	0.005	−0.009	0.018	0.004	1.128	1.000
	Voluntary, Imagination	0.009	−0.005	0.023	0.004	2.211	1.000
External, Past	Voluntary, Past	0.004	−0.009	0.018	0.004	1.093	1.000
	Spontaneous, Imagination	−0.005	−0.018	0.009	0.004	−1.128	1.000
	External, Imagination	3.381e −16	−0.012	0.012	0.004	9.134e −14	1.000
	Voluntary, Imagination	0.004	−0.009	0.018	0.004	1.083	1.000
Voluntary, Past	Spontaneous, Imagination	−0.009	−0.023	0.005	0.004	−2.211	1.000
	External, Imagination	−0.004	−0.018	0.009	0.004	−1.083	1.000

Note. P-value and confidence intervals adjusted for comparing a family of 66 estimates (confidence intervals corrected using the bonferroni method).

Post Hoc Comparisons - Source MW ✻ Orientation

		Mean Difference	95% CI for Mean Difference		SE	t	p_{bonf}
			Lower	Upper			
	Voluntary, Imagination	2.275e −16	−0.012	0.012	0.004	6.146e −14	1.000
Spontaneous, Imagination	External, Imagination	0.005	−0.009	0.018	0.004	1.138	1.000
	Voluntary, Imagination	0.009	−0.005	0.022	0.004	2.231	1.000
External, Imagination	Voluntary, Imagination	0.004	−0.009	0.018	0.004	1.093	1.000

Note. P-value and confidence intervals adjusted for comparing a family of 66 estimates (confidence intervals corrected using the bonferroni method).
